# Differential expression in leaves of *Saccharum* genotypes contrasting in biomass production provides evidence of genes involved in carbon partitioning

**DOI:** 10.1186/s12864-020-07091-y

**Published:** 2020-09-29

**Authors:** Fernando Henrique Correr, Guilherme Kenichi Hosaka, Fernanda Zatti Barreto, Isabella Barros Valadão, Thiago Willian Almeida Balsalobre, Agnelo Furtado, Robert James Henry, Monalisa Sampaio Carneiro, Gabriel Rodrigues Alves Margarido

**Affiliations:** 1 Department of Genetics, University of São Paulo, “Luiz de Queiroz” College of Agriculture, Av Pádua Dias, 11, Piracicaba, 13400-970 Brazil; 2 Department of Biotechnology, Vegetal and Animal Production, Federal University of São Carlos, Center of Agricultural Sciences, Rodovia Anhanguera, km 174, Araras, 13600-970 Brazil; 3 Queensland Alliance for Agriculture and Food Innovation, University of Queensland, Brisbane, 4072 Australia

**Keywords:** Sugarcane, Gene expression, Transcriptomics, RNA-Seq, Polyploid

## Abstract

**Background:**

The development of biomass crops aims to meet industrial yield demands, in order to optimize profitability and sustainability. Achieving these goals in an energy crop like sugarcane relies on breeding for sucrose accumulation, fiber content and stalk number. To expand the understanding of the biological pathways related to these traits, we evaluated gene expression of two groups of genotypes contrasting in biomass composition.

**Results:**

First visible dewlap leaves were collected from 12 genotypes, six per group, to perform RNA-Seq. We found a high number of differentially expressed genes, showing how hybridization in a complex polyploid system caused extensive modifications in genome functioning. We found evidence that differences in transposition and defense related genes may arise due to the complex nature of the polyploid *Saccharum* genomes. Genotypes within both biomass groups showed substantial variability in genes involved in photosynthesis. However, most genes coding for photosystem components or those coding for *phosphoenolpyruvate carboxylases* (PEPCs) were upregulated in the high biomass group. *Sucrose synthase* (SuSy) coding genes were upregulated in the low biomass group, showing that this enzyme class can be involved with sucrose synthesis in leaves, similarly to *sucrose phosphate synthase* (SPS) and *sucrose phosphate phosphatase* (SPP). Genes in pathways related to biosynthesis of cell wall components and *expansins* coding genes showed low average expression levels and were mostly upregulated in the high biomass group.

**Conclusions:**

Together, these results show differences in carbohydrate synthesis and carbon partitioning in the source tissue of distinct phenotypic groups. Our data from sugarcane leaves revealed how hybridization in a complex polyploid system resulted in noticeably different transcriptomic profiles between contrasting genotypes.

## Background

Bioenergy crops are cultivable species with favorable traits as feedstocks for the production of energy [1]. One such biofuel is ethanol, which is produced from the conversion of plant carbohydrates. The disaccharide sucrose is easily converted into ethanol by fermentation, but starch and lignocellulosic polymers have to be converted into monosaccharides prior to fermentation [1, 2]. Lignocellulosic biomass must be disrupted with enzymatic or physical methods as a pretreatment to form a hydrolysable material [2]. Sugarcane culms have been used to produce ethanol from sugar juice fermentation and bagasse, which is also burned to generate electricity. As a result, sugarcane leaves form part of the straw remaining in the field after harvesting. This residual can be used as a biomass source in mills or deposited on the soil to form organic matter. Thus, leaves are a potential biomass supplement to increase the energy supply [3, 4].

Sugarcane species are members of the genus *Saccharum*, of the Poaceae family. There are two ancestral species, *S. robustum* and *S. spontaneum*. The former was the ancestor of the cultivated *S. officinarum* and *S. edule* [5, 6]. Other two cultivated species, *S. barberi* and *S. sinense*, are derived from crosses between *S. officinarum* and *S. spontaneum* [5, 6]. Genotypes of *S. officinarum* were used for cultivation due to their high capacity to produce and store sucrose. Sugarcane stalks are the primary source of sucrose for industrial purposes and have historically been the main target of breeding efforts [7]. Later, crosses of *S. officinarum* with *S. spontaneum* were proposed to avoid abiotic and biotic stresses. Recently, breeding programs have directed efforts to obtain more fibrous genotypes - the so-called energy canes. Because wild genotypes show substantial variability [8, 9], they can be used as a source to introgress traits such as fiber content and stalk number, increasing total biomass yield [10].

Modern sugarcane breeding can benefit from a molecular framework to unravel the underlying genetic basis of important traits. Polyploidy is an inherent characteristic of the *Saccharum* genomes, with *S. officinarum* presenting 80 chromosomes (2n = 8x = 80) and ancient genotypes with a large chromosome number variation [11]. More than 80% of the chromosomes of modern hybrids come from *S. officinarum*, 10–20% from *S. spontaneum* and the remaining are recombinants. There is also aneuploidy in the homeologous groups [12]. The high ploidy in cultivars results in a complex genome of 10 Gbp, that can be represented by an x = 10 monoploid genome [6]. Despite this genomic complexity, progress has been achieved in understanding the role of proteins in carbon partitioning to sucrose or cell wall. Several studies have investigated gene expression to improve understanding of changes in pathways among different plant parts. This has identified the expression of enzymes involved in sucrose metabolism [13, 14], like *sucrose synthase*, that can show organ-specific expression patterns [15, 16]. The expression of genes coding proteins related to cellulose, hemicellulose and lignin metabolism was explored by comparing genotypes contrasting in biomass or in cell wall-related traits [17, 18]. Genes coding for enzymes of the lignin pathway were stimulated in a high-biomass genotype [18], and their expression levels were higher in bottom rather than top internodes [17]. Singh and colleagues [19] found that high-biomass genotypes of an F2 population were more photosynthetically active, as a result of the upregulation of genes coding for photorespiration, Calvin cycle and light reaction proteins.

A wide range of functional categories have been found in studies of gene expression in sugarcane leaves including transporter activity, regulation, response to stimulus and to stress [14, 20]. In addition to their direct use as a biomass source, leaves are the source tissue with which plants produce photoassimilates used to maintain leaf activities and for cell wall synthesis or sucrose accumulation in vacuoles of the stalks and sink organs [21]. Determining the regulation of genes functionally related to biomass-associated traits has value for potential biotechnological applications [1]. To achieve this, we must enhance our knowledge about genes involved in processes of carbohydrate metabolism, especially those related to production of sucrose and lignocellulosic components. To that end, we evaluated the transcriptomes of twelve diverse sugarcane genotypes divided into two contrasting biomass groups. The broad diversity of these genotypes is reflected by the presence of four *S. spontaneum*, a *S. robustum*, two *S. officinarum* representatives and five hybrid cultivars. The five hybrid cultivars come from different genetic backgrounds, from breeding programs in Argentina, Brazil and the United States. In addition to investigating differential gene expression between the two groups, we aimed to identify biological processes that differed between the genotypes within each group.

## Results

### Data summary

**Fig. 1:**
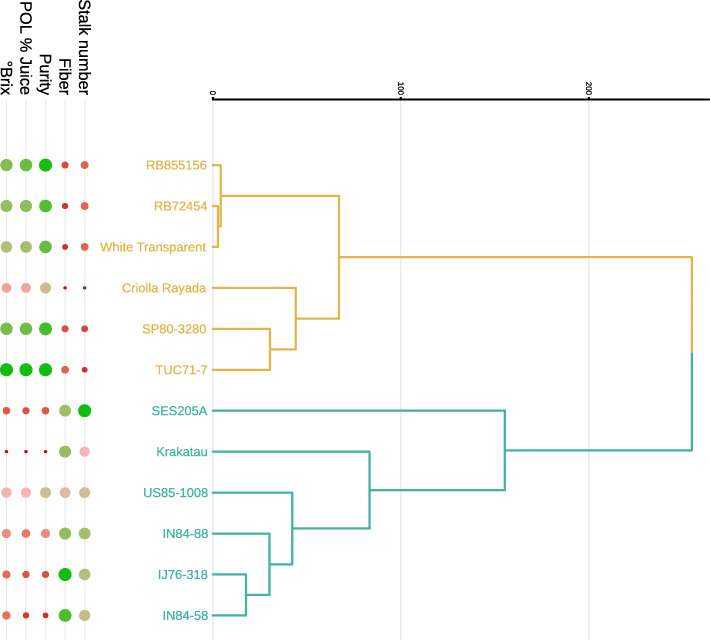
Dendrogram of the twelve sugarcane genotypes based on phenotypic traits. We performed a hierarchical clustering of the genotypes based on Euclidean distances calculated for all evaluated traits. Points at the bottom represent the gradient of the scaled phenotypic measures of each accession, where larger green points represent higher phenotypic values. The measured phenotypic traits include: content of soluble solids in the cane juice (°Brix); polarization or sucrose percentage in the juice (POL % Juice); percentage of sucrose in the total solids of the juice (Purity); percentage of fiber in the bagasse (Fiber); and the number of stalks in each plot

Leaf samples were collected from field-grown plants with six months of age, from twelve different genotypes assigned to two groups contrasting in sucrose-associated traits - soluble solids content, sucrose and purity - and biomass-associated traits - fiber content and number of stalks (Fig. 1 and Additional file 1 - Figure 1). These figures show a group with four *S. spontaneum* representatives - IN84–58, IN84–88, Krakatau and SES205A -, the *S. robustum* genotype IJ76–318 and the hybrid US85–1008. The second group was formed by genotypes that have higher sucrose levels in culms: two *S. officinarum* genotypes - White Transparent and Criolla Rayada -, the hybrid TUC71–7 and more modern hybrids - RB72454, SP80–3280, and RB855156. For simplicity, we will refer to the main difference between the two groups in terms of biomass. Therefore, these genotypes were chosen to include accessions of different *Saccharum* species to form two groups contrasting in biomass content. Although cytogenetic information is limited for sugarcane genotypes, we do expect differences in chromosome numbers and ploidy level among them. Most hybrids, with the exception of US85–1008, have a larger number of *S. officinarum* chromosomes and a minor and variable contribution of *S. spontaneum*, likely with a basic chromosome number of x = 10 [22]. The basic chromosome number of *S. officinarum* is also x = 10, but different numbers have been verified in *S. spontaneum* [22]. Ploidy levels and interspecific hybridization have the potential to affect gene expression patterns, in addition to mechanisms of transcriptional control and epigenetic factors [23, 24]. Nevertheless, our study aimed to find direct associations between transcript abundance and phenotypic traits, without trying to identify the upstream causes of differences in gene expression levels. Our analyses do not depend on prior knowledge about the ploidy of each accession, but we note that variation in chromosome copy counts are possible causes for similarities or differences between particular genotypes.

**Fig. 2:**
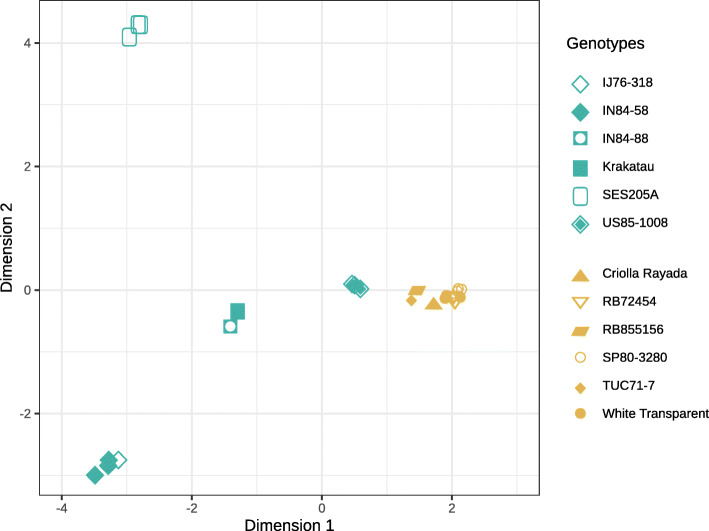
Multidimensional scaling plot to assess dissimilarities between samples. Points in blue represent the high biomass genotypes, while the ones within the low biomass group members are tagged in orange. Different shapes represent different genotypes within each group. Note that three genotypes in each group are represented by three clonal replicates

The mapping rate of sequenced libraries ranged from 80.52 to 85.37% (Table 1 in Additional file 3). To characterize the variability in the expression profiles, we initially assessed the distances between samples based on gene expression levels, using the multidimensional scaling plot to identify clusters. We noted that clonal genotype replicates were close to each other, as expected (Fig. 2). As was the case for phenotypic traits (Fig. 1 in Additional file 1), the first dimension basically separated the high and low biomass groups, and genotypes of the former were farther from each other, revealing higher gene expression variability within the high biomass group. US85–1008 samples clustered between the two groups, apparently reflecting the origin of this genotype in a breeding program. Investigation of the low biomass group (Fig. 2) showed that RB855156 was close to TUC71–7, most likely because it was originated as a hybrid between RB72454 and TUC71–7. In fact, the Brazilian hybrids are closely related, because RB72454 is the offspring of CP53–76 (used as the maternal parent), which is also the maternal grandfather of SP80–3280. The second dimension separated the high biomass genotypes in three sets: i) SES205A at the top; ii) Krakatau, IN84–88 and US85–1008 in the middle; and iii) IN84–58 and IJ76–318. Curiously, in the latter group, an accession classified as *S. robustum* (IJ76–318) grouped closely with a *S. spontaneum* genotype. Variability within the low biomass group is clearly verified if a third dimension is added (Fig. 1 in Additional file 3), in which the most extreme genotypes were RB72454 and SP80–3280 - phenotypically close to each other (Figure 1 in Additional file 1). This result indicates that distances among the low biomass genotypes are smaller than among the high biomass accessions.

We first tested for differences in gene expression levels between the two biomass groups, taking the high biomass group as reference. This resulted in 10,903 downregulated and 10,171 upregulated genes in the low biomass group. In this model, the dispersion estimate includes biological variation between all samples in both groups. This resulted in a biological coefficient of variation (BCV) of 0.86. Although the test within the high biomass group resulted in a BCV of 0.31, more genes were deemed differentially expressed than comparing the groups (Table 2 in Additional file 3). In accordance to the similarity among genotypes, the test within the low biomass group had a similar BCV (0.27) and the lowest number of differentially expressed genes (DEGs) among the three contrasts. Assessing the overlap between these lists of genes, the higher number of unique DEGs occurred when testing for differences among the high biomass genotypes (Figure 2 in Additional file 3), which is consistent with the higher variability among them.

**Fig. 3:**
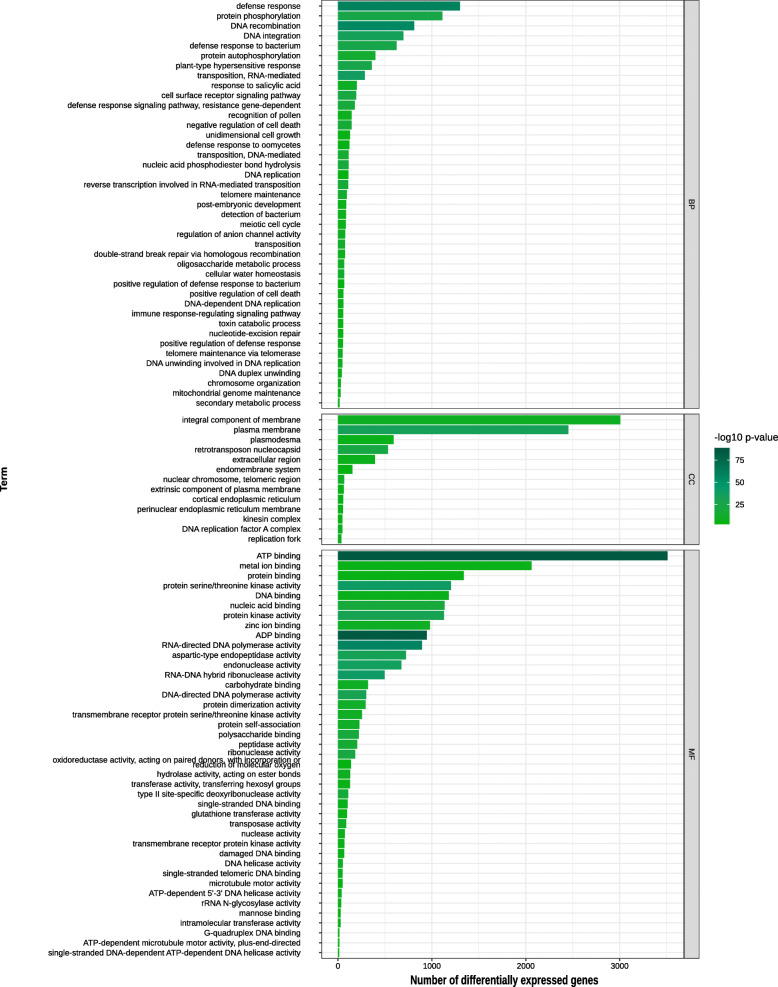
Bar chart of the number of DEGs in each enriched functional class for the differences within the high biomass group. Bars show the number of differentially expressed genes in each Gene Ontology term. Smaller *p*-values are shown by darker green colors. Terms were grouped by the categories BP (Biological Process), CC (Cellular Component) and MF (Molecular Function)

**Fig. 4:**
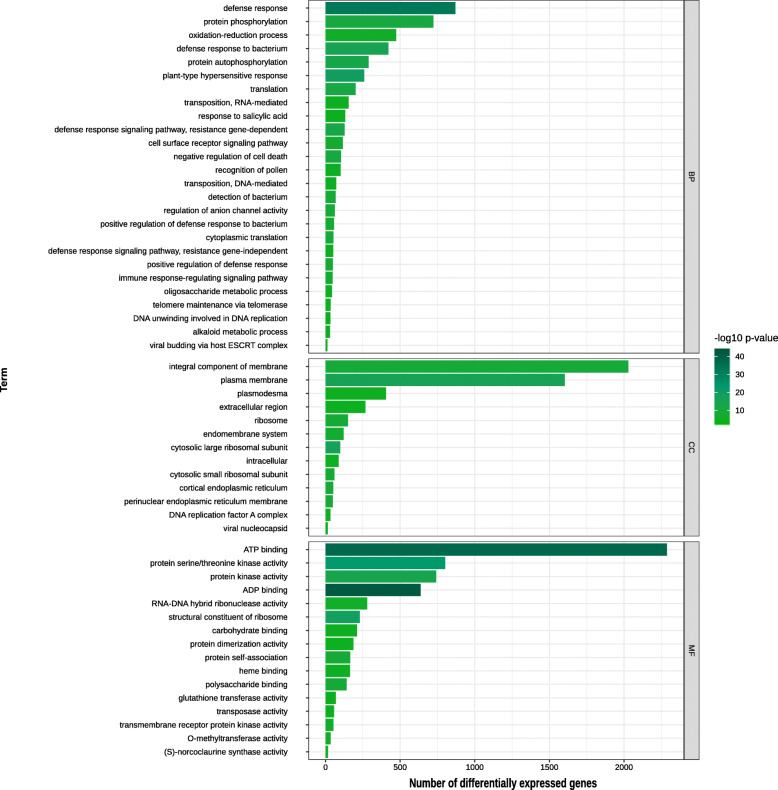
Bar chart of the number of DEGs in each enriched functional class for the differences within the low biomass group. Bars show the number of differentially expressed genes in each Gene Ontology term. Smaller *p*-values are shown by darker green colors. Terms were grouped by the categories BP (Biological Process), CC (Cellular Component) and MF (Molecular Function)

Enrichment analysis was used to assess if functional categories are overrepresented among DEGs, giving evidence of widespread changes in the transcriptional landscape of biological pathways. Functional enrichment analysis with DEGs from the comparison between biomass groups revealed changes in translation and DNA integration – which is a parent term of transposon integration in the Gene Ontology (GO) hierarchy (Figure 3 in Additional file 3). The tests comparing genotypes within the two groups showed many enriched GO terms related to transposition, defense-related and carbohydrate-related (Figs. 3 and 4). Differential expression of transposition-associated genes was more marked when contrasting the two biomass groups and within the high biomass genotypes (Figure 4 in Additional file 3). Also, the high biomass genotypes showed significant differences in the expression level of genes related to cell division, replication and post-replication repair terms. On the other hand, in addition to DEGs related to replication, transcription and kinases, the test within the low biomass group revealed differences in *O-methyltransferase activity* (Figure 4 in Additional file 3). The molecular function *glutathione transferase activity* was enriched in both within-group contrasts (Figs. 3 and 4). We also found changes in genes coding for proteins involved in the response to salicylic acid in both tests.

**Fig. 5:**
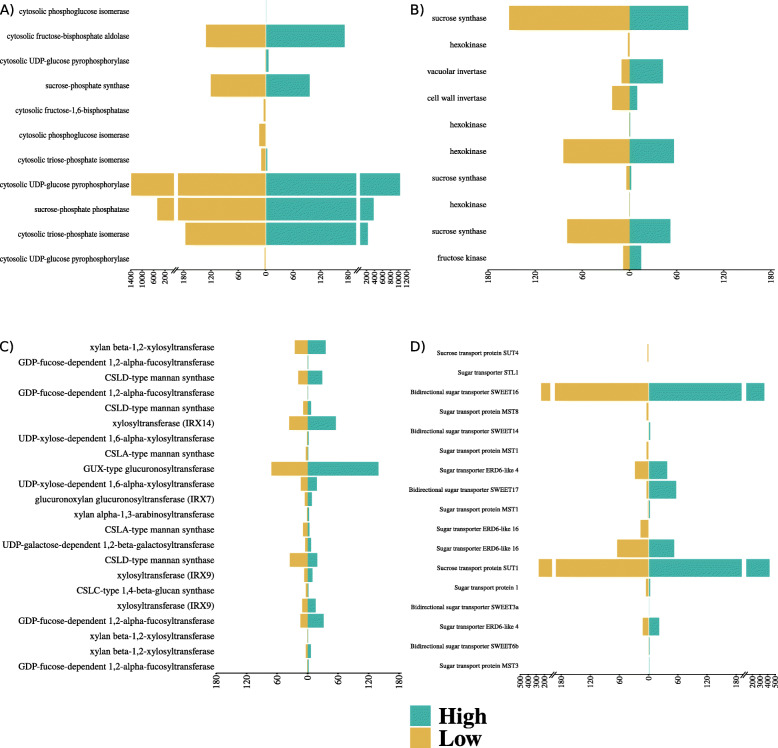
Expression of DEGs involved with sucrose metabolism: synthesis (**a**); degradation (**b**); synthesis of cell wall compounds (**c**); and sucrose and sugar transporters (**d**). Gene expression in each biomass group was calculated using the mean of the normalized counts per million. Note that the scale is different among plots. The high biomass group is colored in blue (right side) and the low biomass group in orange (left side)

**Fig. 6:**
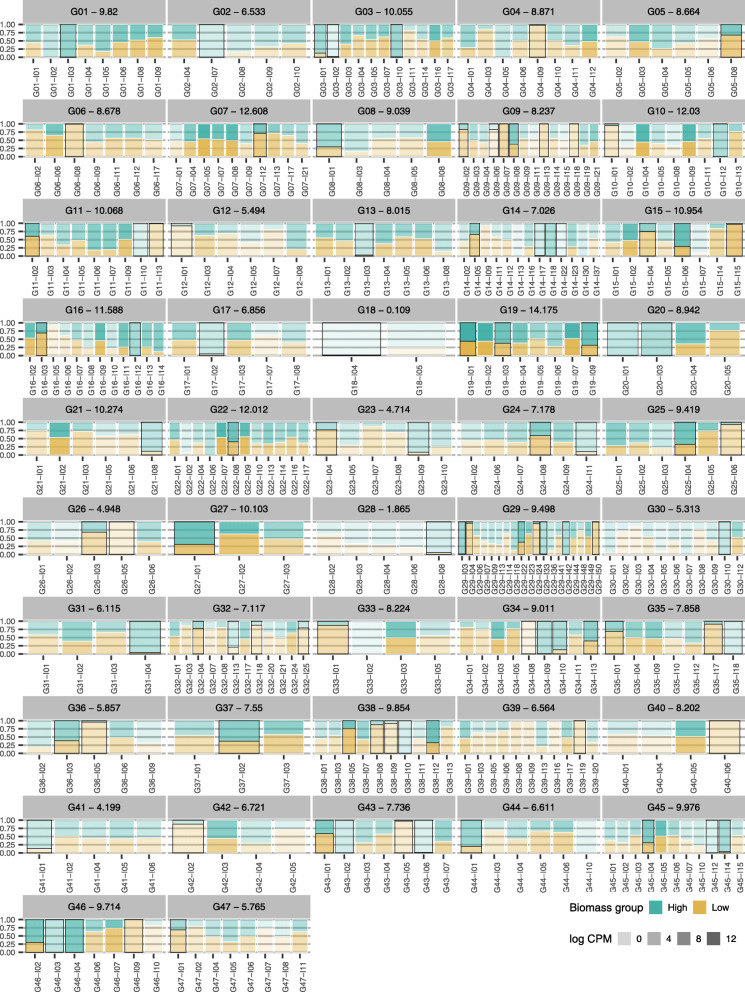
Expression profiles of differentially expressed transcripts of photosynthesis-related genes. Differential expression at the gene level was not significant for the corresponding genes. For each isoform, bar lengths correspond to the relative expression levels in each biomass group. Color intensity represents the logarithm of the counts per million (cpm) of the corresponding transcript. For each gene identifier we also show the log2 of the average counts per million. Differentially expressed transcripts are indicated by black edges

A functional enrichment test performed with the common DEGs detected in the three contrasts corroborates defense response and transposition, as well as gives evidence of a possible genomic stress (Figure 5 in Additional file 3). Using the 7350 DEGs in the pairwise intersection of within-groups contrasts, enrichment analysis revealed changes in the synthesis of cell wall (Figure 6 in Additional file 3).

### Co-expressed genes and metabolic pathways

We identified 16 modules with co-expressed genes, with the number of genes in each module ranging from 514 to 7814. Functional analyses among annotated co-expressed genes in each set revealed enriched GO terms in eleven of these modules (Table 3 in Additional file 3). We identified an overlap of translation- and transcription-related terms predominantly in modules one and seven, such as those involved in the assembly of ribosomal subunits, protein processing, protein degradation and processing of RNAs (Table 3 and Figure 7 in Additional file 3).

Cellular components of chloroplasts were found in five modules of the network: three, seven, eight, eleven and sixteen (Table 3 in Additional file 3). Module 16 was mostly formed by genes related to chloroplast, photosystem and photosynthesis (Figure 7 in Additional file 3). This was the only module to show enrichment of responses to hormones (abscisic acid, cytokinin, ethylene and gibberellin) and these DEGs were mainly repressed in high biomass genotypes (Figure 8 in Additional file 3). We noticed that many genes in module 16 showed high absolute log fold change (LFC) values in all three contrasts, but to a lesser extent in the comparison between *S. officinarum* and the low biomass hybrids (Figure 9 in Additional file 3). This is explained by the expression profile of the genes present in this module, for which the expression level in the low biomass group was higher and similar among the samples (Figure 10 in Additional file 3).

The results of the comparison between the main groups identified up and downregulated DEGs in all metabolic processes provided by the MapMan4 functional BINs (Figure 11 in Additional file 3). Many genes involved in photophosphorylation were downregulated in the low biomass group, annotated as components of the *photosystem II (Psb) proteins*, *photosystem I (Psa)* and *cytochrome (Pet) subunits* and *photosystem I assembly* (YCF3 and YCF4) (Figure 12 in Additional file 3). Other genes of the photosynthesis light reactions were differentially expressed within the two groups, in both cases consistently upregulated in the genotypes with the lowest fiber content (Figure 13 and Figure 14 in Additional file 3). However, genes coding for proteins acting on C4/CAM photosynthesis were downregulated in White Transparent (Figure 14 in Additional file 3). This is in accordance with our co-expression analysis, where many photosynthesis genes with high LFC were present in low biomass genotypes and in US85–1008, but were non-DE when White Transparent was compared to low biomass hybrids (Figure 9 in Additional file 3). DEGs coding for *phosphoenolpyruvate carboxylase* (PEPC) were repressed in low biomass genotypes, being expressed at similar levels in the high biomass accessions (Figure 15 in Additional file 3).

Compared to the high biomass group, low biomass genotypes showed lower expression of genes related to secondary metabolism, such as those annotated to the monolignol synthesis (Figure 16 in Additional file 3). However, the MapMan4 lignin pathway revealed upregulation of certain enzymes in the low biomass genotypes: *phenylalanine ammonia lyase* (PAL), *caffeic acid O-methyltransferase* (COMT), *4-coumarate: CoA ligase* (4CL), *cinnamyl-alcohol dehydrogenase* (CAD) and a *β-glucosidase* (Figure 17 in Additional file 3). US85–1008 and the wild *S. spontaneum* genotypes were similar in the expression of genes coding for enzymes of the lignin metabolism, with significant differences for five genes - a 4CL, a *β-glucosidase*, a *Caffeoyl-CoA O-methyltransferase* and two *cinnamoyl-Coa reductases* (CCR) (Figure 18 in Additional file 3).

We observed that many genes coding for enzymes acting on xylan were upregulated in high biomass genotypes, even in the within-group comparisons (Fig. 5c and Additional file 3 - Figure 19). Regarding cell modification and degradation, a 1*,6-alpha-xylosidase* was highly expressed in the low biomass group (Figure 19-B in Additional file 3). Genes annotated with *xylosyltransferase activity* were co-expressed with those involved with the Golgi apparatus, membrane components and endocytosis, being more highly expressed in high biomass genotypes (Table 3 - Module 10 and Figure 10 in Additional file 3). This is expected given that the Golgi apparatus synthesizes most polysaccharides of the cell wall, where transferases catalyze the synthesis of the xyloglucan backbone and side branches [25]. We also found significant differences in the expression levels of genes associated with cell wall flexibility. In particular, DEGs coding for *expansins* of the β subfamily were more highly expressed in *S. spontaneum* and *S. robustum* (Figure 20 in Additional file 3).

The biomass groups revealed different expression levels of genes coding for enzymes of sucrose metabolism. *Sucrose-phosphate synthase* (SPS) and *sucrose-phosphate phosphatase* (SPP) genes were upregulated in low biomass genotypes (Fig. 5a). Curiously, genes coding for *sucrose synthase* (SuSy) - an enzyme family mainly involved with sucrose degradation - were upregulated in the low biomass group and in US85–1008 (Fig. 5b and Additional file 3 - Figure 21). The comparison between groups also showed different expression levels of genes coding for sucrose transport proteins SUT1 and SUT4. Although SUT4 was strongly upregulated in the low biomass group (Figure 22 in Additional file 3), SUT1 was highly expressed in the high biomass genotypes (Fig. 5d). We found different expression profiles of genes coding for sugar transporters of the same family. Genes coding for SWEETs (*Sugars will eventually be exported transporters*) were downregulated in the low biomass group, while within the groups these DEGs showed a genotype-specific expression (Figure 22-B in Additional file 3).

### Assessing gene expression at different levels

We evaluated how processes are functionally enriched according to the quantification method grouping counts at the gene or transcript level, considering only the contrast between the two main biomass groups. For both approaches, around 30% of each reference set (transcripts or genes) passed the minimum expression threshold (Table 1 in Additional file 4). For 5886 DEGs, none of their corresponding individual transcripts showed statistically significant evidence of differential expression. On the other hand, 8693 genes showed at least one DET, but were not differentially expressed when read counts were gathered at the gene level (Figure 1 in Additional file 4). In addition to the six functional terms enriched among DEGs, analysis of differentially expressed transcripts (DETs) revealed enrichment of another 44 terms (Table 3 in Additional file 4). *Geranylgeranyl-Diphosphate Geranylgeranyltransferase* enrichment indicates changes in the synthesis of geranylgeranyl, a precursor of chlorophyll, carotenoids and gibberellins via the 2-C-methyl-D-erythritol 4-phosphate pathway. This is reinforced by the enrichment of *phytoene synthase*, acting on geranylgeranyl diphosphate in the carotenoid synthesis pathway. We also found enrichment of enzymes acting on precursors of sterols, in the isoprenoid biosynthesis pathway: *farnesyl-diphosphate farnesyltransferase activity* and *squalene synthase activity*. Two non-DEGs coding for *glyceraldehyde-3-phosphate dehydrogenases* (GAPDH) showed five DETs, and the DET with the higher expression level was upregulated in high biomass genotypes (Figure 4 in Additional file 4). Enrichment of GAPDH activity can likely be associated to the photosynthetic carbon reduction promoted by this enzyme, because we found DETs annotated as chloroplastic GAPDHs (Figure 4 in Additional file 4).

Combining the expression levels of DETs to obtain gene-level quantifications can result failure to detect DEGs, masking important functional changes. As an example, we considered the annotated genes of the *photosynthesis* biological process. We found five DEGs without any corresponding DETs – in fact, individual transcripts for three of these genes did not pass the expression filter, due to their low expression level (Figure 3 in Additional file 4). At the same time, 47 non-DE genes revealed at least one DET (Fig. 6). Lowly expressed isoforms did show significant differential expression when the fold changes were very high, i.e., when expression occurred almost entirely in one of the biomass groups (Fig. 6).

## Discussion

Clustering based on gene expression profiles grouped samples in accordance to their phenotypic measures, but also revealed differences within the groups. A higher BCV when contrasting groups was expected because we used different genotypes as replicates of the same group (Fig. 2). The two within-group contrasts are relevant to capture differences between hybrids and wild genotypes that present similar phenotypes. Previously, using SSR genotyping of a subset of the Brazilian Panel of Sugarcane Genotypes, TUC71–7 and SP80–3280 were assigned to the same subpopulation, RB72454 and RB855156 to another and, separately, White Transparent and IN84–58 to the two remaining subpopulations [26]. Indeed, the third dimension of the multidimensional scaling based on gene expression showed that SP80–3280 clustered apart from RB72454 (Fig. 1 in Additional file 3). We hypothesize that the lower number of DEGs in the low biomass group reflects sugarcane breeding, because the hybrids in this group have a higher genomic contribution from *S. officinarum*. Hence, they are not only phenotypically more similar to Criolla Rayada and White Transparent than the high biomass accessions, but also share similar gene expression profiles.

The position of accession US85–1008 between the biomass groups also seemingly reflects the sugarcane breeding history, because this hybrid diverged from the high biomass genotypes more than *S. officinarum* (Criolla Rayada and White Transparent) did from commercial hybrids. Furthermore, the high biomass group included US85–1008 and accessions of two ancestral species – *S. spontaneum* and *S. robustum*. Samples of the *S. spontaneum* SES205A were grouped apart, possibly reflecting the diversity within the subpopulations of this species [8]. The wild sugarcane genotypes of the high biomass group showed substantial differences in their expression profiles and we did not find any evidence of kinship among them in the scientific literature. Wild genotypes, particularly those of *S. spontaneum*, have specific alleles that make them a source of variability for sugarcane breeding. Based on SSR markers, IN84–58 showed more species-specific fragments than Badila and Ganda Cheni - *S. officinarum* and *S. barberi* genotypes, respectively [26]. Also, IN84–58 showed a similar expression profile to IJ76–318, a *S. robustum* accession. In fact, Ferreira and colleagues [27] concluded that *S. spontaneum* and *S. robustum* can have similar expression patterns and group together, separately from *S. officinarum* or a hybrid accession.

Transposition-associated terms were enriched among DEGs both for between- and within-group comparisons. Phylogenetically close species have different transposable elements (TEs) families and differ in the number of TEs in the genome [28]. *Saccharum* species have a high number of TEs, mainly Long Terminal Repeat (LTR) retrotransposons [29, 30]. We suggest that the differential expression of TEs was likely due to the genome differences among the genotypes compared in each contrast. *S. officinarum* showed less differential expression of transposition-related genes in comparison to hybrids relative to that found in the comparisons between groups or between US85–1008 and the other high biomass genotypes (Figure 4 in Additional file 3). This may partly be explained by the higher contribution of the *S. officinarum* genome in hybrids and by large differences between the genomes of the wild canes. This is reinforced by the observation that the divergence between *S. officinarum* and *S. spontaneum* is partially due to the expansion of two TE families in *S. officinarum* [31]. TEs may demonstrate restricted expansion in specific genomes, such as certain families of miniature inverted-repeat transposable elements (MITE) with proliferation-specificity to the *T. aestivum* subgenomes [32]. Moreover, the activity of TEs resulting from polyploidization is analogous to the induction of TEs promoted by stresses [28], a form of genomic shock [33, 34], which is a well described phenomenon in allopolyploids [35]. We can conclude that differences in transposition found within the low biomass group were largely due to variation between commercial hybrids and White Transparent, similar to the observation when contrasting *S. officinarum* to the cultivar RB867515 [27].

Polyploidy creates an imbalance in the nucleotide pool, causing genomic stress in the cell and triggering non-additive expression of genotype-specific responsive genes and other stochastic differences [36, 37]. In addition to polyploidy, hybridization is also a potential cause of genetic variation leading to changes in gene expression between hybrids and parental genotypes. In Asteraceae, Qi and colleagues identified hybridization as the main cause for non-additive expression after comparing gene expression levels of parents (*Chrysanthemum nankingense* and *Tanacetum vulgare*), the interspecific hybrid and three derived allopolyploids [24]. Along with transposition, we noted enriched defense-associated terms when comparing both biomass groups (Figs. 3 and 4). There is evidence that proteins involved in basal metabolism can be more active during stresses. For instance, Ferreira et al. [27] hypothesized that upregulation of histone genes in a hybrid genotype arose from changes in epigenetic control caused by the genomic stress of hybridization. Carson and colleagues [14] evaluated gene expression in sugarcane leaves and found, among many functions, genes coding for proteins responsible for the maintenance and control of cellular metabolism, as well as transport and stress responses. Not only does ploidy regulate these responses, but genes coding for resistance proteins were also upregulated in culms to protect against the stress caused by increased sugar levels in sucrose-rich genotypes [38]. Genotypes in the high biomass group differed in their response to oxidation-reduction, presenting changes in genes whose products are associated to detoxification. *Glutathione transferases*, involved in detoxification, display gene classes occurring in tandem on plant genomes, coding for enzymes acting over a wide range of substrates [39]. Previously, higher expression levels of transcripts related to glutathione-S-transferase were observed in a fiber-rich genotype [18].

The co-expression analysis complemented the enrichment tests based on sets of DEGs. Genes associated with transposition formed two clusters of co-expressed genes that showed similarities within the groups (Table 3 and Figure 10 in Additional file 3). The machineries of replication, transcription, translation and regulatory mechanisms were enriched with similarly expressed genes. Our differential expression analysis involved leaf samples, but no carbon assimilation terms were enriched among DEGs. Interestingly, genes whose products are involved with this process were grouped in a co-expressed module (Table 3 in Additional file 3). Depending on the contrast assessed, pathway analysis showed changes in specific photosynthesis processes, such as C4/CAM photosynthesis and photorespiration (Figures 11, 13 and 14 in Additional file 3). Recently, Singh and colleagues [19] detected upregulation of almost all photosynthesis-related coding genes in high biomass genotypes. As a C4 grass, sugarcane photosynthesis includes a pathway to obtain a four-carbon compound, a process that occurs in the mesophyll and is orchestrated by PEPC. In agreement with Verma and colleagues [40], we noted that high biomass genotypes may require a more intense expression of PEPC coding genes to support metabolic functions other than sucrose accumulation. Expression of PEPC genes was lower in young leaves associated with maturing culms but was practically invariable in leaves connected with more mature stalks [40]. In addition, a group of photophosphorylation genes coding for *Psa*, *Psb* and cytochrome proteins formed a downregulated cluster in low biomass genotypes (Figure 12 in Additional file 3). The module with photosynthesis co-expressed genes was also enriched with terms related to the responses to four hormones - abscisic acid, cytokinin, ethylene and gibberellin. DEGs annotated with hormone responses inside this co-expression module were downregulated in *S. spontaneum* (Figure 8 in Additional file 3). In fact, Singh and colleagues [19] noted that low fiber sugarcanes showed upregulation of genes involved with responses to auxin, jasmonic acid, salicylic acid, abscisic acid and ethylene [19].

Genes coding for enzymes involved in sucrose synthesis, breakdown and transport had been previously studied in different phenological stages of sugarcane culm development [41] and between varying (groups of) genotypes [17, 18, 38]. The pioneering transcriptome studies in sugarcane addressed gene expression in leaves or leaf rolls [13, 14]. Analysis of tissue-specific expression enabled the detection of functions in leaves and culms [14]. Synthesis of sucrose occurs in sugarcane leaves, followed by its transport through phloem to be stored in stalk parenchyma cells [21]. Clearly, sucrose storage is higher in the hybrids and *S. officinarum* clones analyzed herein (Fig. 1 and Additional file 1 - Table 1). In leaves, higher expression of SPS and SPP coding genes in the low biomass group may indicate that the stalk of these genotypes requires more sucrose. They also showed an upregulated gene coding for *Cell Wall Invertase* (CWINV), an enzyme acting on sucrose hydrolysis and allowing the apoplastic entry of hexoses in the stem parenchyma cell [21]. However, CWINV overexpression can promote monomer accumulation in leaves, impairing carbohydrate storage and affecting growth, as described in cassava [42].

SPS and CWINV have been shown to be highly expressed in sugarcane before maturation of culms, precisely to allow the development of leaves and to compensate for sucrose storage requirements in sink tissue [40]. These authors also pointed out that genes coding for enzymes such as PEPC and SUT1 can show stable or increased expression levels in more mature leaves. Our data shows, that in + 1 leaves, genes coding for SUT4 were upregulated in hybrids and *S. officinarum*. However, the SUT1 coding gene was downregulated in the low biomass group but had a higher overall expression level that SUT4 (Fig. 5d), which makes it difficult to determine which SUTs are more relevant to sucrose accumulation. A gene coding for the SWEET14 protein was described as repressed in *S. officinarum* and *S. spontaneum* [27], but we found a SWEET14 gene repressed in the low biomass group, with no evidence of differential expression within this group. We believe that genes coding sugar transporter proteins or sucrose transporter families may be differentially expressed in a genotype-specific manner (Figure 22-B in Additional file 3).

Carbohydrate metabolism in culms also includes gene products from members of the SuSy family. When differentially expressed in a given contrast, SuSy coding genes were always upregulated in genotypes with the higher sucrose level (Fig. 5b). One DEG was also detected in the two other contrasts; other two DEG coding SuSy were upregulated in US85–1008 (Figure 21-B in Additional file 3). In contrast to its common role in stems, SuSy can synthesize sucrose from the reducing sugars present in leaves. Hoffmann-Thoma and colleagues [43] found a higher SuSy activity than SPS in 60 and 90-day expanded leaves. In the same experiment, they found that the content of hexoses was higher than sucrose and that SPS was more active than SuSy in older leaves (2 through 7). In leaf rolls, a low sucrose breakdown/synthesis ratio indicates that SuSy contributes to sucrose synthesis in young sugarcane tissues [15]. Immature leaf rolls, internodes one to six and roots showed higher expression of SuSy1 than leaves [44]. The same study, however, revealed a highly expressed SuSy2 gene in immature and mature leaf lamina. The five DEGs coding for SuSy identified with Mercator showed low average expression levels in our study (Table 4 in Additional file 3), three of them being upregulated in low biomass genotypes. Thirugnanasambandam and colleagues [16] noted that the expression levels of four SuSy genes in leaves were lower than in other tissues, regardless of genotype. Although SuSy is possibly synthesizing sucrose, we also stress the importance of SPS for sucrose synthesis in the low biomass group (Figure 21-A in Additional file 3).

Genes coding for proteins of the lignocellulose pathways were upregulated in high biomass genotypes. *Expansins* are a class of enzymes that can modify the structure of the cell wall, promoting its expansion [45]. The sugarcane genome has roughly ninety *expansin*-coding genes, mostly from the families *α* and *β* [46]. In Poaceae, *β*-*expansin* members act over the matrix polysaccharides, loosening the cell wall [45]. In our study, the high biomass group showed higher expression of *expansin* genes, possibly promoting the development of the leaf. Because structures of the sugarcane top are relevant as biomass sources for energy cane, leaf growth is a desirable trait. Moreover, wild high biomass canes displayed higher expression of *expansins α* − 2, *β* − 11 and *β* − 3, which can be explored as candidate genes in other functional genomic studies. More directly related to the cell wall, many genes coding enzymes that assemble polysaccharides were upregulated in the high biomass genotypes. We identified genes coding for *xylosyltransferases*, *arabinosyltransferases* and *fucosyltransferases* (Fig. 5c and Additional file 3 – Figure 10 and Figure 19), which are glucosyltransferases involved in the biosynthesis of xyloglucan in the Golgi stacks [25]. Loss of function in a *xylosyltransferase* coding gene led to higher saccharification in mutant rice plants, facilitating xylan extraction [47].

Sugarcane genotypes rich in biomass have a higher content of cellulose, hemicellulose and lignin, in detriment to the sucrose content [48]. Clustering of sugarcane genotypes based on similar biomass and sucrose accumulation traits (Figure 1 in Additional file 1) was confirmed by gene expression (Fig. 2). The high biomass group contained mainly wild genotypes, while the low biomass group was represented by *S. officinarum* and hybrids. The high biomass hybrid US85–1008 is the offspring of a wild female parent - an unknown *S. spontaneum* -, while the low biomass hybrids have other hybrids as female parents [26, 49, 50]. Moreover, the low biomass hybrids we studied are all genetically related, with varying degrees of relatedness. This distinct variability within each of the two groups reflects the genomic differences of the accessions (Figure 1 in Additional file 3). Leveraging wild genotypes in sugarcane breeding can be useful to expand the narrow genetic basis of this crop [49, 51], making it possible to develop cultivars with adequate biomass-associated traits, addressing the current limitations in the field and industry. There are also obstacles in sucrose accumulation, which also have to be taken into account because energy canes must be efficient both in biomass and sugar yields [3].

## Conclusions

This work presented a broad view of the expression of many coding genes in sugarcane leaves of different genotypes. With regard to cell wall, most genes were upregulated in the high biomass group, but in general with low average expression levels. On the other hand, highly expressed genes involved in sucrose synthesis were upregulated in hybrids and *S. officinarum* genotypes. These results agree with current knowledge about the partitioning of carbohydrate to sucrose storage and maintenance of plant structure and metabolism in wild genotypes and modern cultivars. In addition, our research shows that investigating expression profiles in wild genotypes can enhance the understanding of genes selected through domestication and breeding. Expression profiles in other plant parts of wild and cultivated accessions are needed to provide knowledge about the action of the genes involved in carbohydrate metabolism and biomass production. Our data from sugarcane leaves revealed how hybridization in a complex polyploid system resulted in noticeably different transcriptomic profiles between contrasting genotypes.

## Methods

### Plant material

We collected leaves of genotypes from the Brazilian Panel of Sugarcane Genotypes [26], selected from groups contrasting in key biomass traits, as measured by fiber content and stalk number. This panel is managed by the sugarcane breeding program of the Inter-University Network for the Development of the Sugarcane Sector (RIDESA), at the Federal University of São Carlos (Araras, Brazil). No special permission was necessary to collect biological samples from these plants. Genotypes of the high biomass group were IN84–58, IN84–88, Krakatau, SES205A, IJ76–318 and US85–1008. In the low biomass group, we selected White Transparent, Criolla Rayada, TUC71–7, RB72454, SP80–3280 and RB855156. Their phenotypic means for soluble solids content (°Brix), percentage of apparent sucrose present in juice (POL % Juice), purity, fiber content (FIB%) and stalk number are summarized in Table 1 (Additional file 1). We performed a hierarchical clustering and a principal component analysis using these measures, and identified two main groups that reflect the separation of high and low fiber genotypes (Fig. 1 and Additional file 1 - Figure 1).

In the high biomass group, there were four *S. spontaneum* representatives (IN84–58, IN84–88, Krakatau and SES205A), a *S. robustum* (IJ76–318) and a hybrid (US85–1008). SES205A is a genotype from India, used in studies of hybrids generated by crosses with *S. officinarum* [8, 52]. Krakatau is an Indonesian *S. spontaneum* widely used in works about biological nitrogen fixation [8, 53, 54]. Genotypes IN84–88, IN84–58 and IJ76–318 are also from Indonesia, and US85–1008 is an accession originated by a cross between a *S. spontaneum* genotype and US60–313 [8, 50].

Samples of the low biomass group include four hybrid cultivars - TUC71–7, RB72454, SP80–3280 and RB855156 - and two *S. officinarum* genotypes - White Transparent and Criolla Rayada. White Transparent was used during the nobilization process [49, 55]. TUC71–7 is a cultivar from Tucumán-Argentina [26, 49], and RB72454, SP80–3280 and RB855156 are Brazilian commercial hybrids [26].

Replicates of each biomass group consisted in one leaf from each genotype. Additionally, we sampled clonal replicates by collecting three leaves from six genotypes (IN84–58, SES205A, US85–1008, White Transparent, RB72454 and SP80–3280). This resulted in a total of 24 samples – 12 genotypes, half of them with clonal replicates. By doing so, we aimed to sample biological variation at two levels: i) between biomass groups, replicates were composed of different genotypes; ii) clonal replicates of particular genotypes allowed for comparisons within each group. Our goal was to have clonal replicates of distant genotypes within each group.

Portions of the first visible dewlap leaves (+ 1) were collected from six-month-old sugarcane plants in April 2016, grown in the field in Araras, Brazil (22°18′41.0″S, 47°23′05.0″W, at an altitude of 611 m). We collected the middle section of each leaf, removing the midrib. After cutting, they were placed in plastic tubes (50 mL), immediately frozen in liquid nitrogen and stored at − 80 °C until RNA extraction. Figure 1 of Additional file 2 shows a summary of our laboratory and bioinformatics steps.

### RNA extraction, sequencing and quality of the libraries

We used the RNeasy Plant Mini Kit (Qiagen, cat. no. *74904*) with roughly 50 mg of starting leaves to extract total RNA from each sample. RNA quality was evaluated by observing the 25S and 18S rRNAs bands via 1% agarose gel electrophoresis. We assessed RNA integrity via 2100 Bioanalyzer (Agilent Technologies) capillary electrophoresis and only kept samples with RNA Integrity Number (RIN) greater than 8. Libraries were prepared with the TruSeq Stranded kit and sequenced in an Illumina HiSeq 2500 platform. We pooled the 24 libraries and sequenced this pool in two lanes, in paired-end mode (2 × 100 bp).

### Differential expression and functional enrichment analyses

We quantified expression levels of de novo assembled transcripts using Salmon [56] (see Additional file 2 for details about read filtering, de novo transcriptome assembly and functional annotation). Isoform expression information was aggregated to gene-count levels using the tximport R package [57]. Next, the data were filtered for genes with expression levels of at least one count per million (cpm) in at least three samples. We performed differential expression analyses with edgeR [58], using two different strategies. First, all samples were used to design a model with two groups contrasting in biomass content. Next, we fitted two separate models to contrast genotypes within each biomass group, including only the genotypes with clonal replicates of each group in an ANOVA-like test. Two contrasts were performed to obtain a Fold Change value within the groups, comparing US85–1008 with the mean of IN84–58 and SES205A, and White Transparent to the mean of SP80–3280 and RB72454. For each model, the DEGs were those with an FDR-adjusted *p*-value less than 5% [59].

Functional enrichment analyses were performed with the goseq R package [60], separately for each differential expression model. The background set was composed of the expressed genes passing the cpm filter. A GO term was considered enriched among DEGs if its overrepresentation adjusted *p*-value was less than 5%.

Additionally, we carried out tests at the transcript level to find differentially expressed transcripts between the same biomass groups. We then compared the two approaches by measuring the overlap between the lists of DETs and DEGs.

### Co-expression network and gene set enrichment analysis

A co-expression network was built with WGCNA [61], using as input the logarithm of the normalized cpm matrix of the expressed genes. We chose a soft-thresholding power of nine, reaching a correlation coefficient of approximately 0.8 for the scale-free topology fit. Our choice was to build an adjacency matrix preserving the sign of the connection. After hierarchical clustering of genes based on their dissimilarity, modules that were composed of at least 300 genes were considered. We grouped modules that had highly co-expressed genes, using a correlation threshold of 0.75 for the module eigengenes. The sets of genes defined by each module, were used to evaluate the presence of enriched Gene Ontology terms with goseq, again considering an overrepresented adjusted *p-*value less than 5%.

Next, we checked the enrichment of the gene set formed by each co-expression module by ranking genes based on their absolute LFC for each contrast. This analysis was conducted with the GSEAPreranked tool in the GSEA software [62].

### Pathway analysis

The MapMan4 pipeline [63] was used to functionally assign genes to land plant protein categories. The full transcriptome was annotated using the Mercator4 tool. Because the expression quantification was done at the gene level, the transcript identifiers of the Mercator4 mapping file were changed to gene identifiers. Thus, the functional annotation attributed to isoforms of a gene were also combined. Genes in the MapMan4 pathways were tagged and colored based on the LFC from the differential expression tests.

## Supplementary information

**Additional file 1.** Supporting information of the genotypes. Additional tables and figures showing a phenotypic characterization of the genotypes.

**Additional file 2 **Supporting information for methods. Additional information about the methods used in the manuscript: basic statistics and comparison of the de novo assemblies. Annotation of the de novo assembly used as reference*.*

**Additional file 3.** Supporting information for results. Additional tables and figures about the expression analyses: differentially expressed genes in common between the tests, functional enrichment results, co-expression network and data mining of important genes.

**Additional file 4.** Supporting information for differentially expressed transcripts. Additional tables and figures about the analyses comparing the expression of genes and transcripts.

## Data Availability

The raw sequencing data used in this article have been submitted to DDBJ/EMBL/GenBank under the BioProject ID PRJEB38368.

## Abbreviations

4CL: 4-coumarate: CoA ligase
BCV: Biological coefficient of variation
CAD: Cinnamyl-alcohol dehydrogenase
CCR: Cinnamoyl-Coa reductases
COMT: Caffeic acid O-methyltransferase
cpm: count per million
CWINV: Cell Wall Invertase
DEGs: Differentially expressed genes
DETs: Differentially expressed transcripts
GAPDH: Glyceraldehyde-3-phosphate dehydrogenases
GO: Gene Ontology
LFC: Log fold change
LTR: Long Terminal Repeat
MITE: Miniature inverted-repeat transposable elements
PAL: Phenylalanine ammonia lyase
PEPC: Phosphoenolpyruvate carboxylase
Psa: Photosystem I
Psb: Photosystem II
SPP: Sucrose-phosphate phosphatase
SPS: Sucrose-phosphate synthase
SuSy: Sucrose synthase
SUT: Sucrose transport protein
SWEET: Sugars will eventually be exported transporters
TE: Transposable element
