# Supplementary material for: Differential expression in leaves of Saccharum genotypes contrasting in biomass production provides evidence of genes involved in carbon partitioning
Source: BMC Genomics. 2020 Sep 29;21:673. doi: 10.1186/s12864-020-07091-y (PMC7526157; doi:10.1186/s12864-020-07091-y)
Supplement: Supplementary file 1 — Additional file 1. Supporting information of the genotypes. Additional tables and figures showing a phenotypic characterization of the genotypes. [file 12864_2020_7091_MOESM1_ESM.pdf]

S1 - Supporting information of the genotypes

Phenotypic characterization

We obtained measures of °Brix, POL % Juice, purity, fiber and stalk number of four replicates per genotype during two harvest years - 2015 and 2016. In 2015 the phenotypic data was collected from plant cane, while ratoon cane was used for the phenotypic measures in 2016. The mean of the measures for each genotype are shown in Table 1. Using the dissimilarity of the genotypes based on euclidean distances of the phenotypic measures, we performed a hierarchical clustering to obtain a dendrogram. Samples clustered in two main groups, reflecting the separation of high and low fiber genotypes (Figure **??**). We observed the same clustering pattern through a principal component analysis (Figure 1), where the first dimension explained more than 90% of the variation. These groups differed in their biomass content, in terms of fiber and stalk number, and by their sucrose accumulation in culms, which are related with POL % Juice, °Brix and purity.

Table 1: Phenotypic measures of the genotypes. °Brix corresponds to the content of soluble solids in the cane juice. POL % Juice is the polarization measurement of the sucrose percentage in the juice. Purity indicates the percentage of sucrose in the total solids of the juice. Fiber is the percentage of fiber in the bagasse and stalk number represents the number of stalks in each plot.

Genotype	°Brix	POL % Juice	Purity	Fiber	Stalk number
Criolla Rayada	16.53 ± 1.37	12.79 ± 1.82	77.05 ± 4.76	9.83 ± 1.21	22.71 ± 14.44
White Transparent	19.6 ± 2.02	17.15 ± 2.54	87.12 ± 4.31	10.82 ± 1.54	115.88 ± 24.64
RB72454	20.52 ± 1.66	18.03 ± 2.38	88.69 ± 3.15	11.14 ± 2.08	117.62 ± 13.03
RB855156	21.01 ± 1.19	19.15 ± 1.51	91.07 ± 3.17	12 ± 1.07	115.38 ± 20.85
SP80-3280	21.29 ± 1.88	19.1 ± 2.3	89.5 ± 3.02	12.14 ± 0.68	78.12 ± 23.17
TUC71-7	22.76 ± 0.8	20.77 ± 0.77	91.25 ± 0.39	12.97 ± 1.27	47.88 ± 14.96
US85-1008	17.61 ± 1.11	13.55 ± 1.54	76.81 ± 6.17	18.96 ± 1.82	298.71 ± 72.87
SES205A	13.99 ± 3.14	8.77 ± 3.78	59.89 ± 16.62	21.86 ± 3.6	464.62 ± 91.42
Krakatau	12.03 ± 2.02	6.53 ± 2.09	53.08 ± 9.52	22.22 ± 3.5	244.38 ± 61.53
IN84-88	15.91 ± 2.43	10.64 ± 4.24	66.03 ± 22.63	22.35 ± 2.26	353.86 ± 62.8
IN84-58	14.78 ± 1.89	7.88 ± 1.5	55.28 ± 4.86	24.64 ± 1.7	316.88 ± 105.6
IJ76-318	14.54 ± 3.24	8.78 ± 3.49	58.52 ± 11.12	25.55 ± 4.03	334.14 ± 117.65

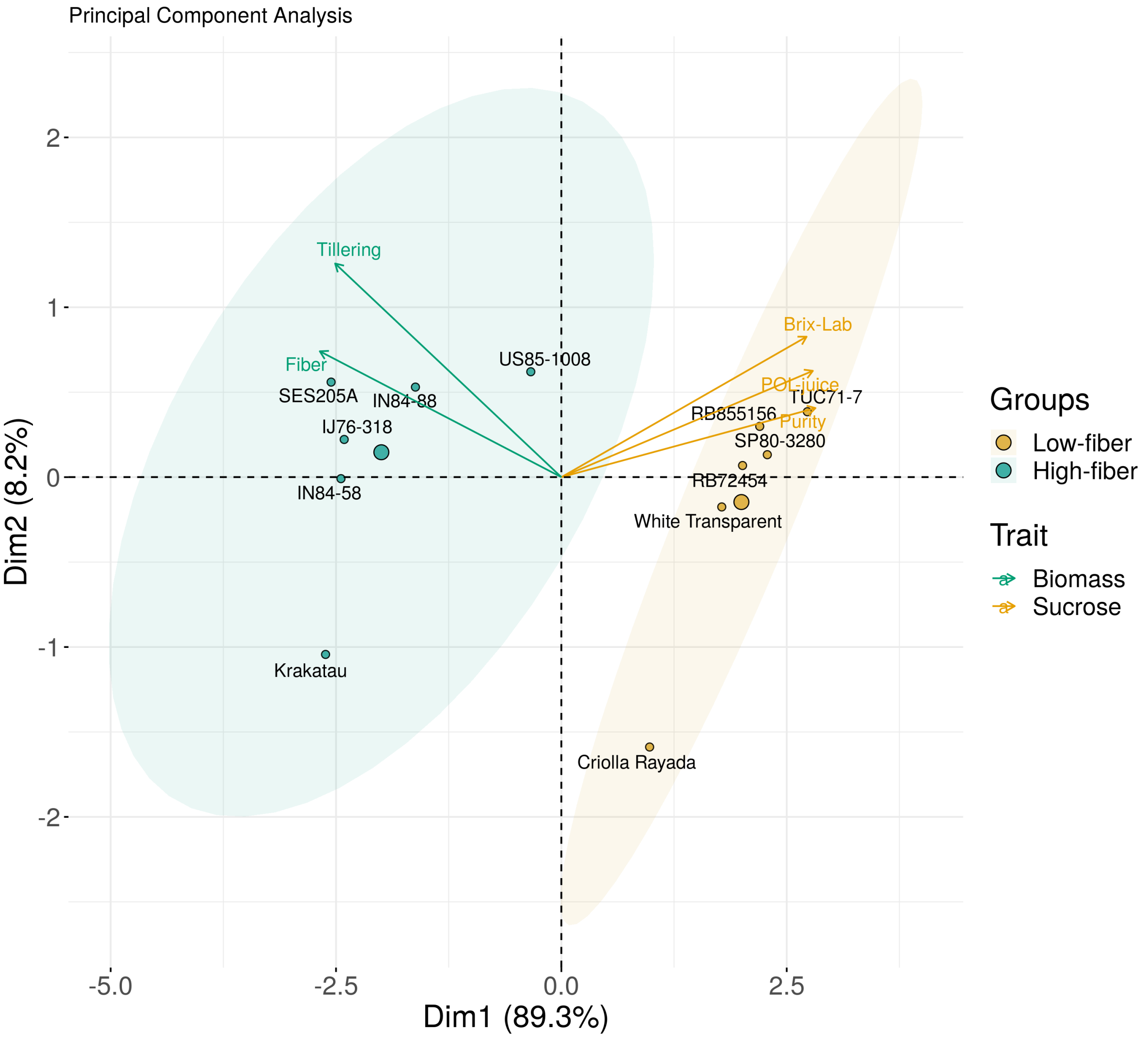

Figure 1: **Principal component analysis of biomass and sucrose traits** Yellow projections reflect traits related to higher biomass, while green projetions indicate traits associated to sucrose accumulation. The two biomass groups (red and blue) were found based on the k-means algorithm.
