# Supplementary material for: Differential expression in leaves of Saccharum genotypes contrasting in biomass production provides evidence of genes involved in carbon partitioning
Source: BMC Genomics. 2020 Sep 29;21:673. doi: 10.1186/s12864-020-07091-y (PMC7526157; doi:10.1186/s12864-020-07091-y)
Supplement: Supplementary file 2 — Additional file 2 Supporting information for methods. Additional information about the methods used in the manuscript: basic statistics and comparison of the de novo assemblies. Annotation of the de novo assembly used as reference. [file 12864_2020_7091_MOESM2_ESM.pdf]

S2 - Supporting information for methods

1 Methods summary

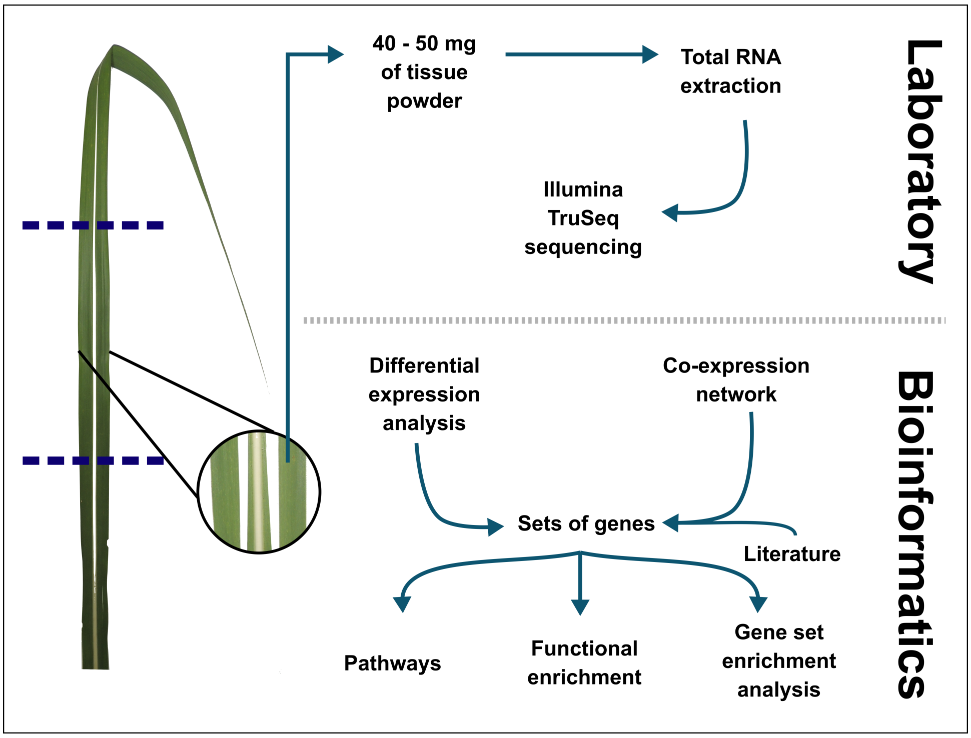

Figure 1: **Summary of the main methodological steps** Workflow is divided in laboratory and bioinformatic steps. The laboratory section includes steps from the collection of leaves to the Illumina TruSeq sequencing. The bioinformatics section indicates the analyses to find gene sets, differential expression and co-expression networks, including the search for genes in literature. From the sets of genes, we searched for pathway mappings in MapMan and functional enrichment of Gene Ontology terms.

2 Preprocessing of raw reads and *de novo* transcriptome assembly

To evaluate the quality of sequencing runs, we used the diagnosis tool FASTQC [1]. Removal of adapters and low quality bases was performed with Trimmomatic [2], using windows with a minimum average Phred quality score of 20. We also trimmed the first 12 bases and kept reads with at least 75 bases.

We performed transcriptome *de novo* assemblies with Trinity (v.2.8.4) [3], using as parameters the k-mer size of 25, normalization of FASTQ pairs (*normalize_by_read_set*) and minimum contig length (*min_contig_length*) of 300. In addition to these these parameters, in the second assembly we set k-mer coverage (*min_kmer_cov*) to two. In the third assembly we set the maximum number of reads to combine into a single path (*max_reads_per_graph*), minimum percent identity (*min_per_id_same_path*) and maximum differences between two paths (*max_diffs_same_path*) to 3,000,000, 90 and 10, respectively.

This means that we increased the number of reads anchored within a graph, reduced the identity for the paths be combined into a single one and allowed more differences to combine two paths. A fourth *de novo* transcriptome was built combining parameters of the two previous assemblies.

Assembly statistics, such as the number of unigenes and number of transcripts, are in Table 1. The completeness of the *de novo* assemblies was evaluated with BUSCO [4] using the set of longest isoforms of the assembly and datasets of conserved orthologs from *Viridiplantae* and *Liliopsida.* To assess RNA-Seq read representation, we mapped the preprocessed reads to each transcriptome using HISAT [5]. This mapping was used only as a metric to assess the assembly with the best read representation.

Table 1: ***De novo* transcriptome assembly statistics.** Assembly 1 was *de novo* assembled with the common set of parameters. A k-mer coverage of at least two was used for the Assembly 2. Assembly 3 had as parameters regarding a same path: a maximum of 3,000,000 reads to be combined, a minimum identity of 90% and up to 10 differences. All the parameters of the assemblies 2 and 3 were combined to generate Assembly 4.

	**Assembly 1**	**Assembly 2**	**Assembly 3**	**Assembly 4**
**Total trinity genes**	174,755	111,670	166,517	106,010
**Total trinity transcripts**	437,123	331,229	373,896	279,896
**Percent GC**	48.94	49.29	48.63	49.03
**'Genes' N50**	1,734	1,779	1,926	1,902
**Longest isoform N50**	1,123	1,325	1,192	1,396

Mapping of reads to the longest isoform was higher in both the first and second assemblies (Table 2). The representation of complete conserved orthologs (Table 3) was higher in the first assembly, particularly for the full *Viridiplantae* gene set.

Table 2: **Number of input reads and overall alignment rate.** Assembly 1 was *de novo* assembled with the common set of parameters. A k-mer coverage of at least two was used for the Assembly 2. Assembly 3 had as parameters regarding a same path: a maximum of 3,000,000 reads to be combined, a minimum identity of 90% and up to 10 differences. All the parameters of the assemblies 2 and 3 were combined to generate Assembly 4.

Sample	Input fragments	Assembly 1	Assembly 2	Assembly 3	Assembly 4
Criolla Rayada	17,449,229	74.80	74.43	73.08	72.72
IJ76-318	20,673,607	74.27	73.84	72.75	72.44
IN84-58	26,880,400	73.53	73.49	72.05	71.58
IN84-58	17,388,508	73.50	73.34	71.94	71.75
IN84-58	19,386,319	74.41	74.02	72.62	72.35
IN84-88	16,343,061	73.47	72.86	72.02	71.68
Krakatau	18,943,919	73.52	73.14	72.41	71.98
RB72454	15,828,991	73.77	73.31	72.31	72.31
RB72454	16,474,182	74.00	73.62	72.69	72.44
RB72454	20,096,595	74.59	74.25	73.30	73.03
RB855156	19,062,736	74.66	74.19	73.43	73.03
SES205A	17,771,779	74.55	74.66	73.47	73.22
SES205A	19,574,309	73.78	73.57	72.45	72.21
SES205A	19,234,085	73.14	72.91	71.77	71.56
SP80-3280	15,332,802	74.40	74.11	72.75	72.57
SP80-3280	16,877,418	74.16	73.78	72.44	72.51
SP80-3280	22,863,504	75.06	74.66	73.48	73.18
TUC71-7	18,759,428	75.32	75.07	73.96	73.61
US85-1008	22,047,957	73.88	73.78	72.74	72.31
US85-1008	21,531,366	71.69	71.56	70.70	70.74
US85-1008	16,146,634	74.94	74.94	74.16	73.82
White Transparent	16,157,056	74.13	73.88	72.14	72.43
White Transparent	18,175,403	74.20	73.64	71.91	72.48
White Transparent	17,786,061	75.07	74.75	73.60	73.30

Because the first and second assemblies showed the best results for these two criteria, we evaluated their DETONATE RSEM-EVAL Score. This model-based score is based on support of RNA-Seq reads and other factors, such as assembly compactness [6]. The first assembly score (-4.42×10^9) was higher than that of the second assembly (-16.91×10^9).

Finally, we examined the full-length transcript counting using the script *analyze_blastPlus_topHit_coverage.pl* comparing our two assemblies with UniProt. After aligning the transcripts of each assembly with UniProt proteins by Blastx, we grouped Blast hits using the script *blast_outfmt6_group_segments.tophit_coverage.pl*. For all protein coverage thresholds, the number of proteins was higher in the first assembly (Figure 2). This analysis also indicates that the first assembly was more appropriate for the subsequent steps of the analysis.

Using the complete transcriptome obtained with the first assembly, 97.4% of conserved eukaryotic orthologs were found as complete (Table 4). The assembled transcriptome proves to be a suitable sugarcane reference, representing the eukaryotic orthologs as well as other sugarcane transcriptomes used as references [7].

Table 3: **Percentage of conserved orthologs from *Viridiplantae* and *Liliopsida* present in the longest isoform assemblies.** Assembly 1 was *de novo* assembled with the common set of parameters. A k-mer coverage of at least two was used for the Assembly 2. Assembly 3 had as parameters regarding a same path: a maximum of 3,000,000 reads to be combined, a minimum identity of 90% and up to 10 differences. All the parameters of the assemblies 2 and 3 were combined to generate Assembly 4.

	**Assembly 1**	**Assembly 2**	**Assembly 3**	**Assembly 4**
**Viridiplantae**				
Complete and single-copy BUSCOs (S)	74.4	69.1	63.3	68.8
Complete and duplicated BUSCOs (D)	1.2	1.4	1.9	0.9
Fragmented BUSCOs (F)	17.4	21.9	24.9	22.8
Missing BUSCOs	7.0	7.6	9.9	7.5
**Liliopsida**				
Complete and single-copy BUSCOs (S)	68.4	67.7	62.0	65.2
Complete and duplicated BUSCOs (D)	2.2	2.0	2.0	1.6
Fragmented BUSCOs (F)	17.4	17.5	21.0	18.8
Missing BUSCOs	12.0	12.8	15.0	14.4

3 Transcriptome annotation

We performed annotation with TRINOTATE [9], using: i) homology search of our sequences to the UniProt database; ii) protein domain identification from Pfam; iii) prediction of protein signal peptides and transmembrane domains. This approach can recover information from the databases of Gene Ontology (GO), Kyoto Encyclopedia of Genes and Genomes (KEGG) and eggNOG.

References

[1] Simon Andrews. FastQC: a quality control tool for high throughput sequence data. *Available in: http://www.bioinformatics.babraham.ac.uk/projects/fastqc*, 2010.

[2] Anthony M. Bolger, Marc Lohse, and Bjoern Usadel. Trimmomatic: A flexible trimmer for Illumina sequence data. *Bioinformatics*, 30(15):2114–2120, 2014.

[3] Manfred G Grabherr, Brian J Haas, Moran Yassour, Joshua Z Levin, Dawn A Thompson, Ido Amit, Xian Adiconis, Lin Fan, Raktima Raychowdhury, Qiandong Zeng, Zehua Chen, Evan Mauceli, Nir Hacohen, Andreas Gnirke, Nicholas Rhind, Federica di Palma, Bruce W Birren, Chad Nusbaum, Kerstin Lindblad-Toh, Nir Friedman, and Aviv Regev. Full-length transcriptome assembly from RNA-Seq data without a reference genome. *Nature Biotechnology*, 29(7):644–652, jul 2011.

[4] Felipe A. Simão, Robert M. Waterhouse, Panagiotis Ioannidis, Evgenia V. Kriventseva, and

	**Complete Transcriptome**	**Longest Isoforms**
Complete and single-copy BUSCOs (S)	17.2	69.0
Complete and duplicated BUSCOs (D)	80.2	22.1
Fragmented BUSCOs (F)	2.0	7.9
Missing BUSCOs	0.6	1.0

Table 4: Percentage of conserved orthologs from *Eukaryota* present in the complete transcriptome and in the longest isoforms.

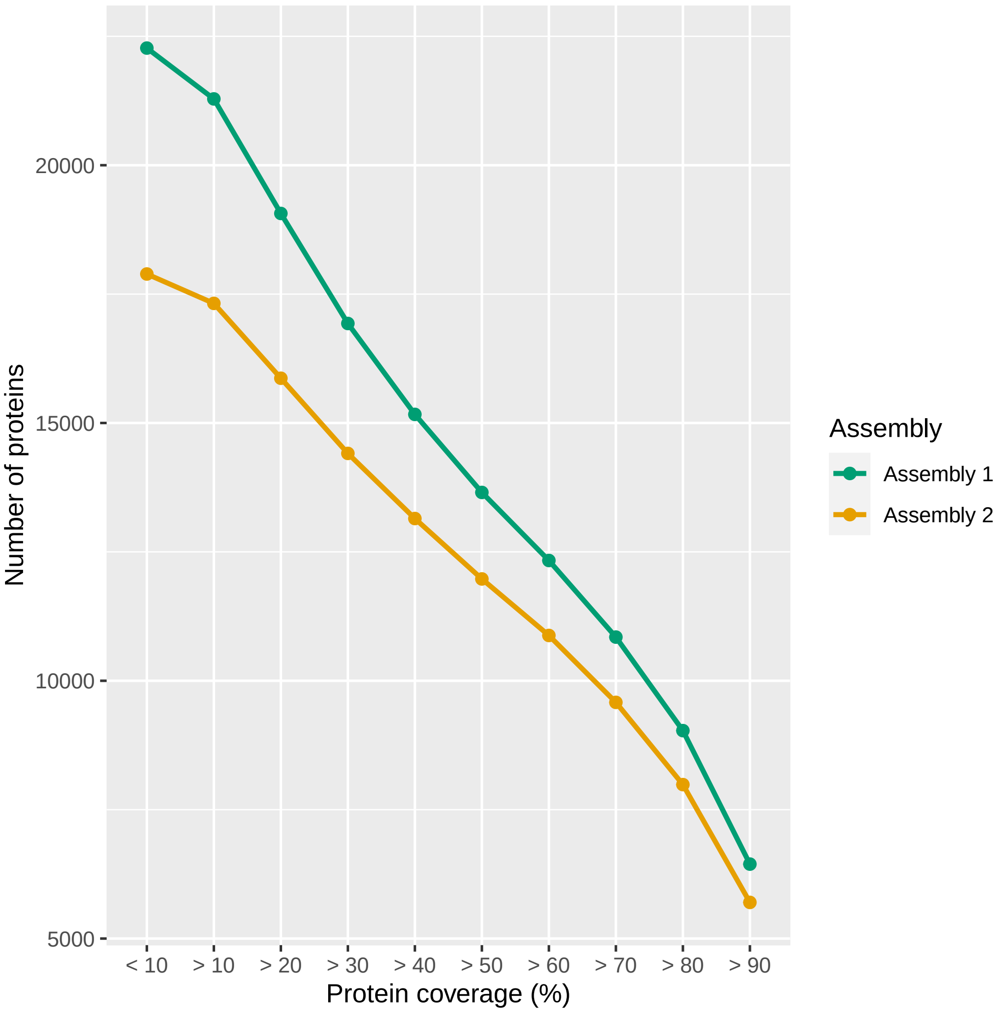

Figure 2: Counts of full-length transcripts for varying thresholds of protein coverage.

Evgeny M. Zdobnov. BUSCO: assessing genome assembly and annotation completeness with single-copy orthologs. *Bioinformatics*, 31(19):3210–3212, oct 2015.

[5] Daehwan Kim, Ben Langmead, and Steven L Salzberg. HISAT: a fast spliced aligner with low memory requirements. *Nature Methods*, 12(4):357–360, mar 2015.

[6] Bo Li, Nathanael Fillmore, Yongsheng Bai, Mike Collins, James A. Thomson, Ron Stewart, and Colin N. Dewey. Evaluation of de novo transcriptome assemblies from RNA-Seq data. *Genome Biology*, 15(12):1–21, 2014.

[7] Nam V. Hoang, Agnelo Furtado, Virginie Perlo, Frederik C. Botha, and Robert J. Henry. The Impact of cDNA Normalization on Long-Read Sequencing of a Complex Transcriptome. *Frontiers in Genetics*, 10, jul 2019.

[8] Claudio Benicio Cardoso-Silva, Estela Araujo Costa, Melina Cristina Mancini, Thiago Willian Almeida Balsalobre, Lucas Eduardo Costa Canesin, Luciana Rossini Pinto, Monalisa Sampaio Carneiro, Antonio Augusto Franco Garcia, Anete Pereira De Souza, and Renato Vicentini. De novo assembly and transcriptome analysis of contrasting sugarcane varieties. *PLoS ONE*, 9(2):e88462, feb 2014.

[9] Donald M. Bryant, Kimberly Johnson, Tia DiTommaso, Timothy Tickle, Matthew Brian Couger, Duygu Payzin-Dogru, Tae J. Lee, Nicholas D. Leigh, Tzu-Hsing Kuo, Francis G. Davis, Joel

Bateman, Sevara Bryant, Anna R. Guzikowski, Stephanie L. Tsai, Steven Coyne, William W. Ye, Robert M. Freeman, Leonid Peshkin, Clifford J. Tabin, Aviv Regev, Brian J. Haas, and Jessica L. Whited. A Tissue-Mapped Axolotl De Novo Transcriptome Enables Identification of Limb Regeneration Factors. *Cell Reports*, 18(3):762–776, jan 2017.
