# Supplementary material for: Differential expression in leaves of Saccharum genotypes contrasting in biomass production provides evidence of genes involved in carbon partitioning
Source: BMC Genomics. 2020 Sep 29;21:673. doi: 10.1186/s12864-020-07091-y (PMC7526157; doi:10.1186/s12864-020-07091-y)
Supplement: Supplementary file 3 — Additional file 3. Supporting information for results. Additional tables and figures about the expression analyses: differentially expressed genes in common between the tests, functional enrichment results, co-expression network and data mining of important genes. [file 12864_2020_7091_MOESM3_ESM.pdf]

S3 - Supporting information for results

1 Mapping and quantification

Mapping rates of preprocessed reads obtained with Salmon [1] are shown in Table 1.

Table 1: Salmon mapping rates of the preprocessed reads. Table contains the percentage of mapping rate for samples in both high and low biomass groups.

Sample	Group	Mapping rate (%)
IJ76-318	High biomass	83.91
IN84-58	High biomass	82.27
IN84-58	High biomass	83.53
IN84-58	High biomass	82.76
IN84-88	High biomass	83.59
Krakatau	High biomass	82.03
SES205A	High biomass	83.00
SES205A	High biomass	84.71
SES205A	High biomass	80.52
US85-1008	High biomass	83.56
US85-1008	High biomass	80.84
US85-1008	High biomass	84.22
Criolla Rayada	Low biomass	84.36
RB72454	Low biomass	83.41
RB72454	Low biomass	82.99
RB72454	Low biomass	83.40
RB855156	Low biomass	84.93
SP80-3280	Low biomass	82.62
SP80-3280	Low biomass	85.37
SP80-3280	Low biomass	84.27
TUC71-7	Low biomass	84.98
White Transparent	Low biomass	83.93
White Transparent	Low biomass	83.65
White Transparent	Low biomass	83.69

2 Differential expression and functional enrichment analyses

2.1 Sample clustering based on expression

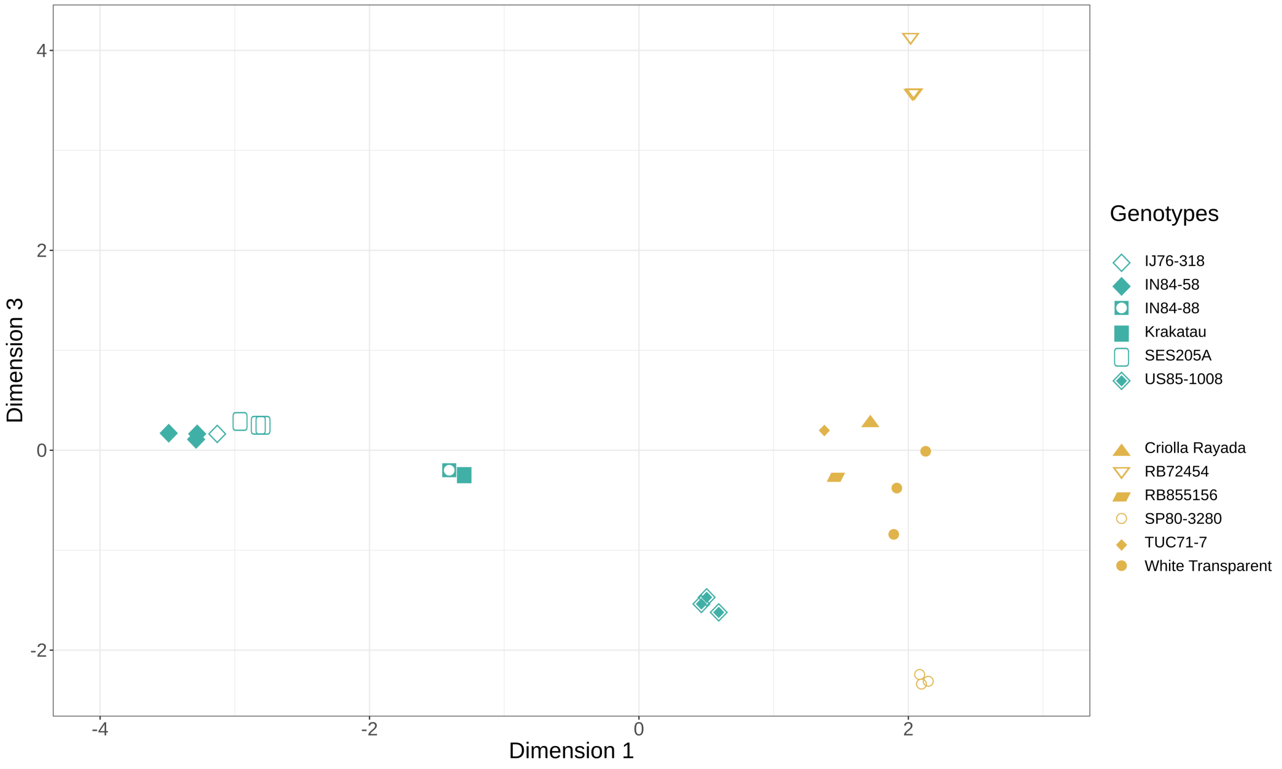

Figure 1: **Multidimensional scaling to assess dissimilarities between samples.** Blue symbols indicate high biomass genotypes, and low biomass genotypes are colored in orange.

2.2 Tests

Table 2: Results of the differential expression analysis in the three proposed tests: i) Low biomass group compared to high biomass group; ii) ANOVA-like test using genotypes within the high biomass group; iii) ANOVA-like test using genotypes within the low biomass group.

	**Low biomass vs High biomass**	**ANOVA-like high biomass**	**ANOVA-like low biomass**
Differentially expressed	21074	27981	17099
Not significantly regulated	26602	19695	30577

We evaluated Gene Ontology enriched terms in each of these tests, in the following order:

- Low biomass genotypes compared to the high biomass genotypes (Figure 3)
- Differences within the high biomass group (main document)
- Differences within the low biomass group (main document)

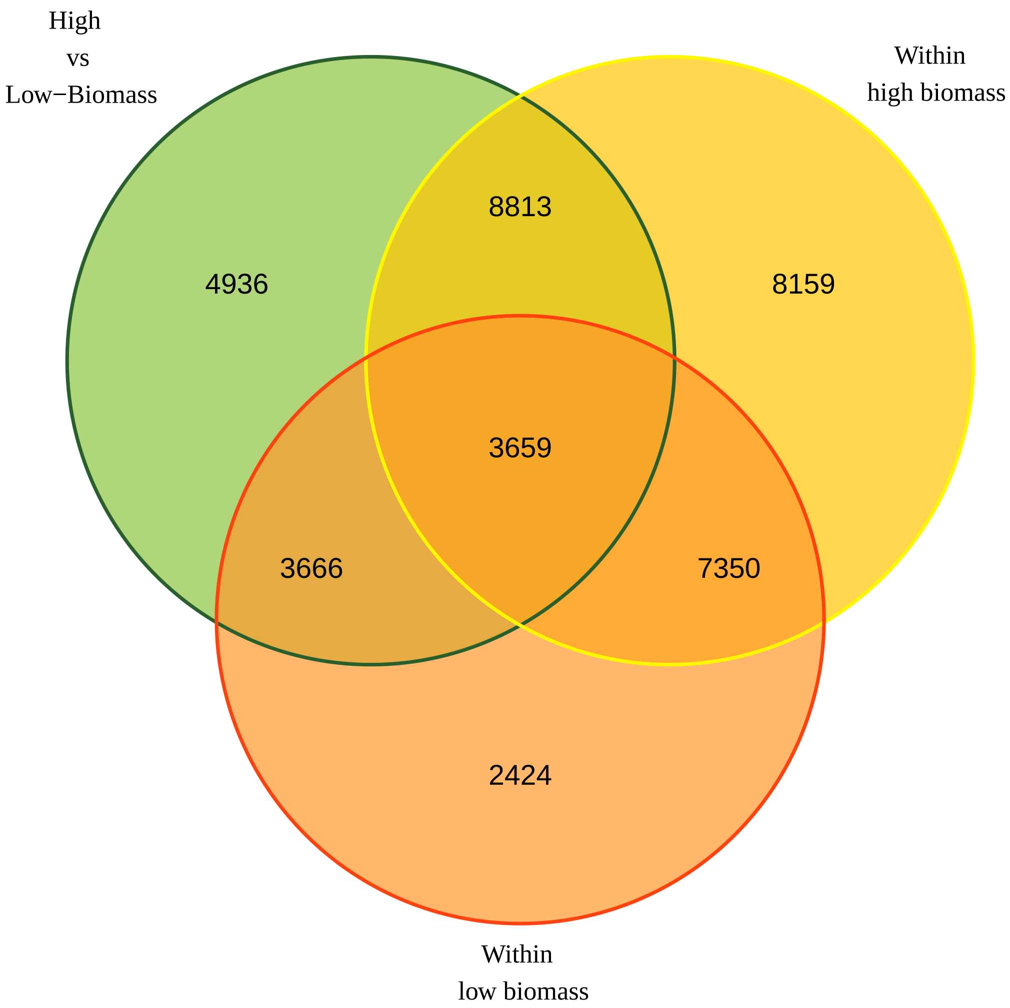

Figure 2: Venn diagram of the overlap between lists of differentially expressed genes in the three tests.

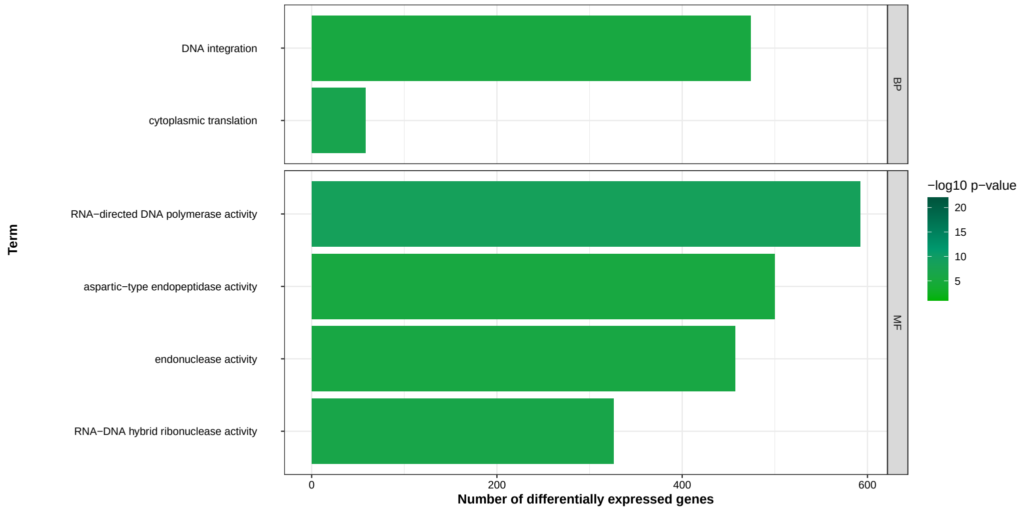

Figure 3: Bar chart of the number of DEGs in each enriched functional class for the biomass group contrast. Gene ontology categories are indicated by BP (Biological Process), CC (Cellular Component) and MF (Molecular Function).

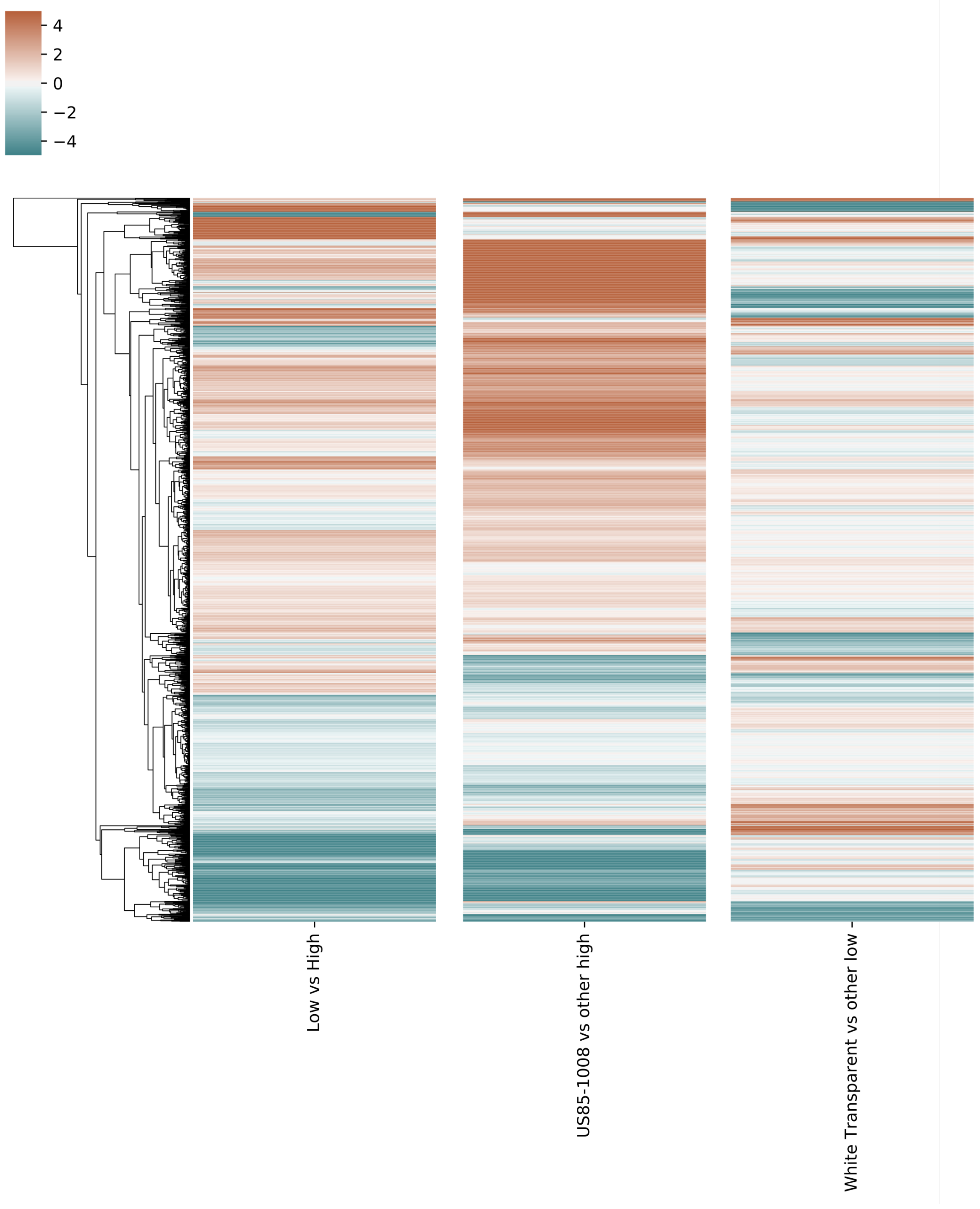

Figure 4: **Heatmap of differential expression for genes associated with transposition.** Each column in the heatmap represent the genes fold changes according to a contrast: (i) *Low vs High* is the comparison between the low biomass to the high biomass group; (ii) *US85-1008 vs other high* represents the comparison of US85-1008 to wild high-fiber canes; (iii) *White Transparent vs other low* is the contrast of White Transparent against the low-fiber hybrids.

Using the common DEGs among the three contrast, the enrichment corroborates differences in stress response and transposition (Figure 5). To avoid the enrichment of these often apparent terms, we performed a functional enrichment analyses using the common genes between the high and low groups contrasts, removing those common to the fiber contrast. With that we verified that sugarcane genotypes, even in a same phenotypic group, have differences in the cell wall biogenesis (Figura 6).

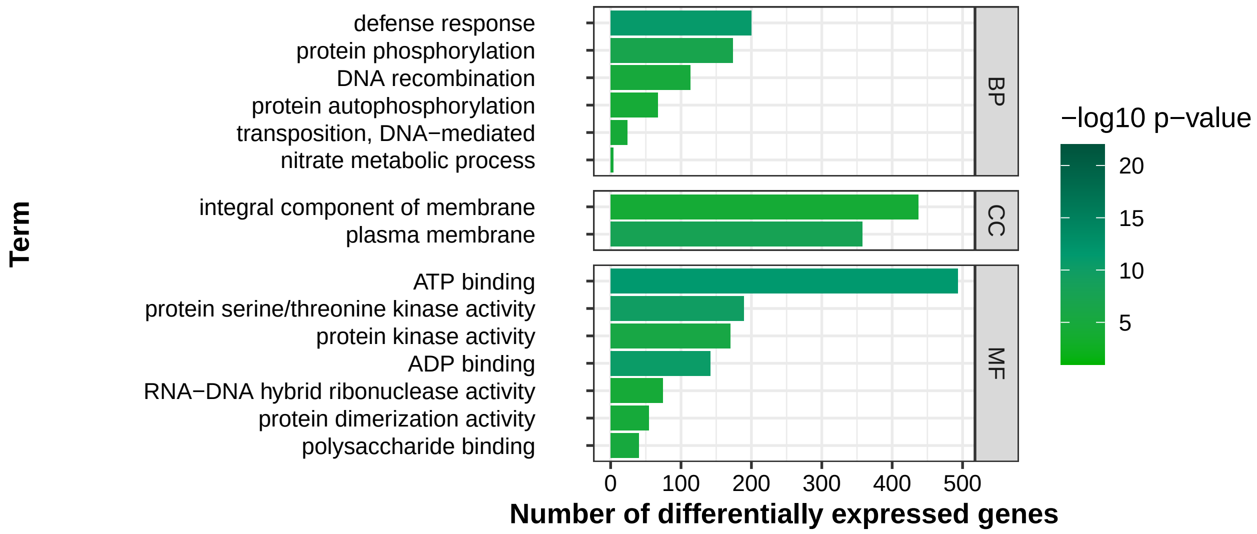

Figure 5: Bar chart of the number of DEGs in each category enriched with common DEGs between the three contrasts. Gene ontology categories are indicated by BP (Biological Process), CC (Cellular Component) and MF (Molecular Function)

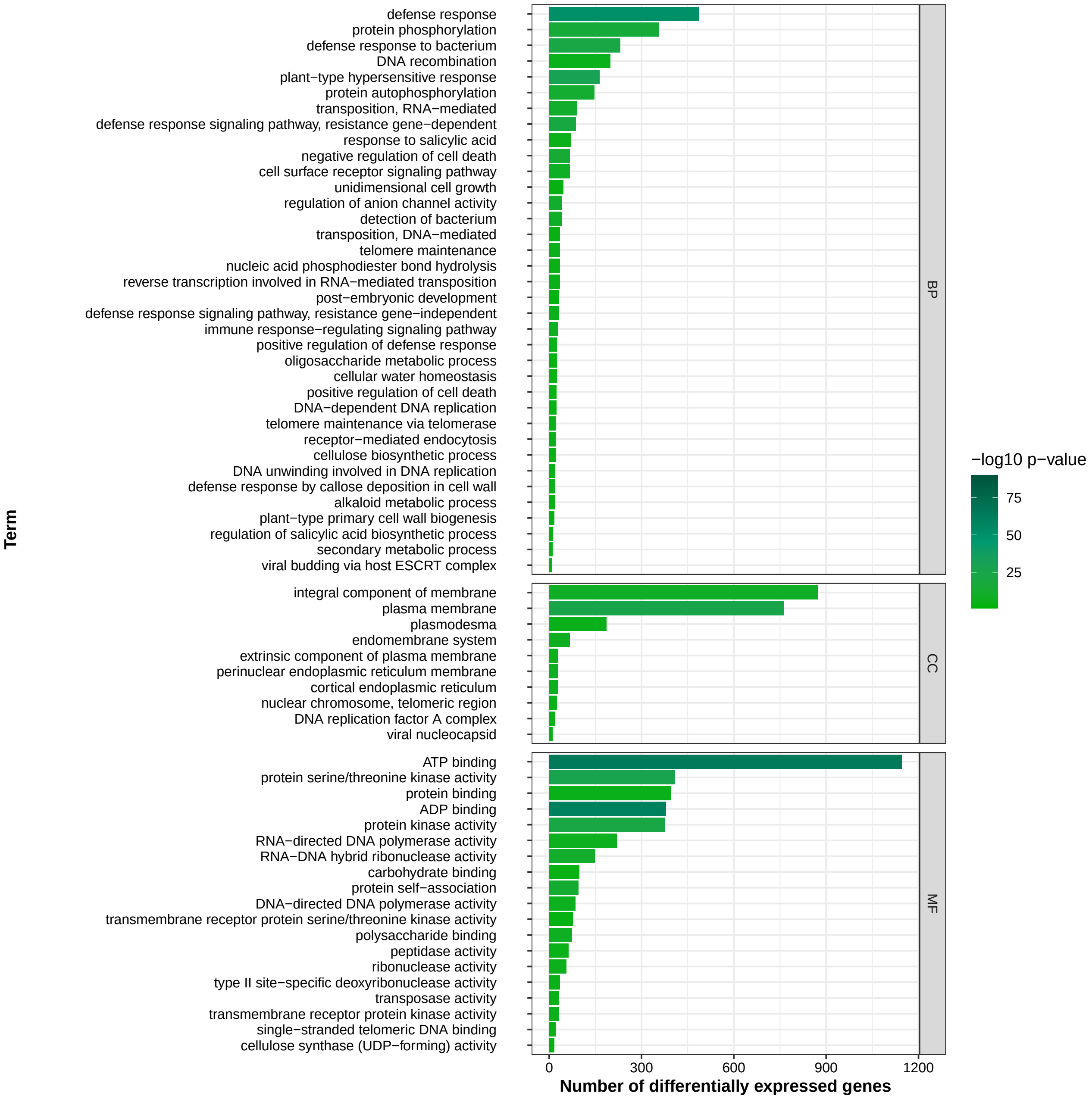

Figure 6: Bar chart of the number of DEGs in each category enriched with common DEGs between the contrasts comparing genotypes within the groups. Gene ontology categories are indicated by BP (Biological Process), CC (Cellular Component) and MF (Molecular Function)

3 Co-expression enrichment

Our co-expression network was built with the genes passing the expression filter. We obtained 16 modules, of which eleven showed enrichment of 289 Gene Ontology terms. Table 3 presents the Gene Ontology terms enriched in each co-expression module.

Table 3: Gene Ontology terms enriched in each co-expression module.

Module	Category	Overrepresented p-value	Term
Module 1	GO:0003735	0.000000000	structural constituent of ribosome
	GO:0006412	0.000000000	translation
	GO:0002181	0.000000000	cytoplasmic translation
	GO:0005840	0.000000000	ribosome
	GO:0022625	0.000000000	cytosolic large ribosomal subunit
	GO:0005829	0.000000000	cytosol
	GO:0005622	0.000000000	intracellular
	GO:0033290	0.000000000	eukaryotic 48S preinitiation complex
	GO:0022627	0.000000000	cytosolic small ribosomal subunit
	GO:0005759	0.000000000	mitochondrial matrix
	GO:0001732	0.000000001	formation of cytoplasmic translation initiation complex
	GO:0016282	0.000000001	eukaryotic 43S preinitiation complex
	GO:0043161	0.000000003	proteasome-mediated ubiquitin-dependent protein catabolic process
	GO:0055114	0.000000004	oxidation-reduction process
	GO:0006099	0.000000018	tricarboxylic acid cycle
	GO:0032153	0.000000021	cell division site
	GO:0005747	0.000000058	mitochondrial respiratory chain complex I
	GO:0005852	0.000000130	eukaryotic translation initiation factor 3 complex
	GO:0030479	0.000000316	actin cortical patch
	GO:0003743	0.000000329	translation initiation factor activity
	GO:0045842	0.000000637	positive regulation of mitotic metaphase/anaphase transition
	GO:0005737	0.000000841	cytoplasm
	GO:0000329	0.000001102	fungal-type vacuole membrane
	GO:0006696	0.000001155	ergosterol biosynthetic process
	GO:0005839	0.000003294	proteasome core complex
	GO:0004298	0.000003823	threonine-type endopeptidase activity
	GO:0016491	0.000004003	oxidoreductase activity
	GO:0043066	0.000004081	negative regulation of apoptotic process
	GO:0010498	0.000004696	proteasomal protein catabolic process
	GO:0006413	0.000005148	translational initiation
	GO:0000502	0.000006289	proteasome complex
	GO:0002183	0.000007142	cytoplasmic translational initiation
	GO:0000272	0.000008485	polysaccharide catabolic process
	GO:0005838	0.000009464	proteasome regulatory particle
	GO:0015986	0.000010074	ATP synthesis coupled proton transport
	GO:0032543	0.000010163	mitochondrial translation
	GO:0010499	0.000012071	proteasomal ubiquitin-independent protein catabolic process
	GO:0000276	0.000014544	mitochondrial proton-transporting ATP synthase complex, coupling factor F(o)
	GO:0006457	0.000015568	protein folding
	GO:0006048	0.000015596	UDP-N-acetylglucosamine biosynthetic process
	GO:0051082	0.000018026	unfolded protein binding

Table 3: Gene Ontology terms enriched in each co-expression module.

Module	Category	Overrepresented p-value	Term
	GO:0034622	0.000023142	cellular macromolecular complex assembly
	GO:0008540	0.000026175	proteasome regulatory particle, base subcomplex
	GO:0022624	0.000029125	proteasome accessory complex
	GO:0006120	0.000032853	mitochondrial electron transport, NADH to ubiquinone
	GO:0004175	0.000036743	endopeptidase activity
	GO:0005743	0.000042846	mitochondrial inner membrane
	GO:0036402	0.000050085	proteasome-activating ATPase activity
	GO:0004099	0.000058786	chitin deacetylase activity
	GO:0006119	0.000066176	oxidative phosphorylation
	GO:0000921	0.000068492	septin ring assembly
	GO:0031105	0.000068492	septin complex
	GO:0032160	0.000068492	septin filament array
	GO:1903475	0.000073496	mitotic actomyosin contractile ring assembly
	GO:0006620	0.000075607	posttranslational protein targeting to endoplasmic reticulum membrane
	GO:0009405	0.000076071	pathogenesis
	GO:0015934	0.000076610	large ribosomal subunit
	GO:0000001	0.000077394	mitochondrion inheritance
	GO:0009062	0.000077940	fatty acid catabolic process
	GO:0004129	0.000087082	cytochrome-c oxidase activity
	GO:0030544	0.000101316	Hsp70 protein binding
	GO:0031072	0.000108075	heat shock protein binding
	GO:0005686	0.000113360	U2 snRNP
	GO:0019878	0.000116452	lysine biosynthetic process via aminoadipic acid
	GO:0045899	0.000122924	positive regulation of RNA polymerase II transcriptional preinitiation complex assembly
	GO:0030234	0.000130228	enzyme regulator activity
	GO:0099132	0.000131513	ATP hydrolysis coupled cation transmembrane transport
	GO:0031204	0.000133465	posttranslational protein targeting to membrane, translocation
	GO:0042254	0.000149007	ribosome biogenesis
	GO:0005685	0.000154174	U1 snRNP
	GO:0034515	0.000164803	proteasome storage granule
	GO:0043248	0.000169156	proteasome assembly
	GO:0000027	0.000183653	ribosomal large subunit assembly
	GO:0000050	0.000193815	urea cycle
	GO:0004753	0.000203954	saccharopine dehydrogenase activity
	GO:0000028	0.000222564	ribosomal small subunit assembly
	GO:0042788	0.000259576	polysomal ribosome
	GO:0005940	0.000324330	septin ring
	GO:0007264	0.000369241	small GTPase mediated signal transduction
	GO:0008541	0.000389399	proteasome regulatory particle, lid subcomplex
	GO:0006032	0.000410471	chitin catabolic process
Module 2	GO:0043531	0.000004750	ADP binding
Module 3	GO:0000943	0.000000001	retrotransposon nucleocapsid
	GO:0008270	0.000000001	zinc ion binding
	GO:0009507	0.000000003	chloroplast
	GO:0009451	0.000000006	RNA modification

Table 3: Gene Ontology terms enriched in each co-expression module.

Module	Category	Overrepresented p-value	Term
	GO:0015074	0.000000012	DNA integration
	GO:0004519	0.000000056	endonuclease activity
	GO:0003964	0.000000066	RNA-directed DNA polymerase activity
	GO:0004190	0.000000083	aspartic-type endopeptidase activity
	GO:0003676	0.000002277	nucleic acid binding
	GO:0006310	0.000003950	DNA recombination
	GO:0009570	0.000027960	chloroplast stroma
	GO:0007004	0.000049015	telomere maintenance via telomerase
	GO:0006261	0.000050414	DNA-dependent DNA replication
Module 4	GO:0043531	0.000000300	ADP binding
	GO:0047268	0.000001561	galactinol-raffinose galactosyltransferase activity
Module 7	GO:0003735	0.000000000	structural constituent of ribosome
	GO:0005730	0.000000000	nucleolus
	GO:0006412	0.000000000	translation
	GO:0005840	0.000000000	ribosome
	GO:0006364	0.000000000	rRNA processing
	GO:0022625	0.000000000	cytosolic large ribosomal subunit
	GO:0003723	0.000000000	RNA binding
	GO:0022627	0.000000000	cytosolic small ribosomal subunit
	GO:0005622	0.000000000	intracellular
	GO:0005739	0.000000000	mitochondrion
	GO:0032040	0.000000000	small-subunit processome
	GO:0005829	0.000000000	cytosol
	GO:0019843	0.000000000	rRNA binding
	GO:0022626	0.000000000	cytosolic ribosome
	GO:0042254	0.000000000	ribosome biogenesis
	GO:0000462	0.000000000	maturation of SSU-rRNA from tricistronic rRNA transcript (SSU-rRNA, 5.8S rRNA, LSU-rRNA)
	GO:0000027	0.000000000	ribosomal large subunit assembly
	GO:0042273	0.000000000	ribosomal large subunit biogenesis
	GO:0000447	0.000000001	endonucleolytic cleavage in ITS1 to separate SSU-rRNA from 5.8S rRNA and LSU-rRNA from tricistronic rRNA transcript (SSU-rRNA, 5.8S rRNA, LSU-rRNA)
	GO:0000480	0.000000001	endonucleolytic cleavage in 5'-ETS of tricistronic rRNA transcript (SSU-rRNA, 5.8S rRNA, LSU-rRNA)
	GO:0051082	0.000000003	unfolded protein binding
	GO:0006457	0.000000007	protein folding
	GO:0000028	0.000000026	ribosomal small subunit assembly
	GO:0000472	0.000000026	endonucleolytic cleavage to generate mature 5'-end of SSU-rRNA from (SSU-rRNA, 5.8S rRNA, LSU-rRNA)
	GO:0008033	0.000000040	tRNA processing
	GO:0030515	0.000000044	snoRNA binding
	GO:0006397	0.000000068	mRNA processing
	GO:0003729	0.000000119	mRNA binding
	GO:0031167	0.000000226	rRNA methylation
	GO:0017056	0.000000229	structural constituent of nuclear pore
	GO:0006414	0.000000286	translational elongation
	GO:0030687	0.000000873	preribosome, large subunit precursor
	GO:0009408	0.000001166	response to heat

Table 3: Gene Ontology terms enriched in each co-expression module.

Module	Category	Overrepresented p-value	Term
	GO:0000049	0.000001168	tRNA binding
	GO:0000463	0.000001366	maturation of LSU-rRNA from tricistronic rRNA transcript (SSU-rRNA, 5.8S rRNA, LSU-rRNA)
	GO:1990904	0.000001859	ribonucleoprotein complex
	GO:0006606	0.000002675	protein import into nucleus
	GO:0034388	0.000003248	Pwp2p-containing subcomplex of 90S preribosome
	GO:0019919	0.000004851	peptidyl-arginine methylation, to asymmetrical-dimethyl arginine
	GO:0000338	0.000005375	protein deneddylation
	GO:0030686	0.000005630	90S preribosome
	GO:0000055	0.000006631	ribosomal large subunit export from nucleus
	GO:0009507	0.000006957	chloroplast
	GO:0005762	0.000008474	mitochondrial large ribosomal subunit
	GO:0035242	0.000008551	protein-arginine omega-N asymmetric methyltransferase activity
	GO:0005654	0.000009141	nucleoplasm
	GO:0008180	0.000010412	COP9 signalosome
	GO:0031429	0.000010913	box H/ACA snoRNP complex
	GO:0005682	0.000011762	U5 snRNP
	GO:0034513	0.000014963	box H/ACA snoRNA binding
	GO:0003899	0.000015136	DNA-directed 5'-3' RNA polymerase activity
	GO:0016282	0.000016113	eukaryotic 43S preinitiation complex
	GO:0006413	0.000016417	translational initiation
	GO:0008168	0.000017298	methyltransferase activity
	GO:0005681	0.000020908	spliceosomal complex
	GO:0004812	0.000020909	aminoacyl-tRNA ligase activity
	GO:0042788	0.000021288	polysomal ribosome
	GO:0034336	0.000021974	misfolded RNA binding
	GO:0060567	0.000021974	negative regulation of DNA-templated transcription, termination
	GO:0008469	0.000022216	histone-arginine N-methyltransferase activity
	GO:0034969	0.000022216	histone arginine methylation
	GO:0003743	0.000026370	translation initiation factor activity
	GO:0006396	0.000026802	RNA processing
	GO:0016554	0.000039355	cytidine to uridine editing
	GO:0000176	0.000042088	nuclear exosome (RNase complex)
	GO:0004386	0.000042349	helicase activity
	GO:0032543	0.000057521	mitochondrial translation
	GO:0001732	0.000061808	formation of cytoplasmic translation initiation complex
	GO:0000398	0.000076151	mRNA splicing, via spliceosome
	GO:0030488	0.000089460	tRNA methylation
	GO:0051117	0.000123216	ATPase binding
	GO:0009536	0.000144513	plastid
	GO:0034511	0.000169454	U3 snoRNA binding
	GO:0032955	0.000200647	regulation of division septum assembly
	GO:0033290	0.000208091	eukaryotic 48S preinitiation complex
	GO:0005736	0.000233738	DNA-directed RNA polymerase I complex
	GO:0042134	0.000243525	rRNA primary transcript binding
	GO:0051028	0.000251357	mRNA transport
	GO:0000469	0.000257832	cleavage involved in rRNA processing

Table 3: Gene Ontology terms enriched in each co-expression module.

Module	Category	Overrepresented p-value	Term
	GO:0005832	0.000258810	chaperonin-containing T-complex
	GO:0006418	0.000300001	tRNA aminoacylation for protein translation
	GO:0003871	0.000302662	5-methyltetrahydropteroyltriglutamate-homocysteine S-methyltransferase activity
	GO:0010157	0.000325097	response to chlorate
	GO:0004328	0.000335979	formamidase activity
	GO:0006383	0.000337829	transcription from RNA polymerase III promoter
	GO:0030295	0.000367275	protein kinase activator activity
	GO:0009295	0.000368216	nucleoid
	GO:0031118	0.000385301	rRNA pseudouridine synthesis
	GO:0016811	0.000392026	hydrolase activity, acting on carbon-nitrogen (but not peptide) bonds, in linear amides
	GO:0070181	0.000408561	small ribosomal subunit rRNA binding
	GO:0005732	0.000425031	small nucleolar ribonucleoprotein complex
	GO:0043021	0.000440732	ribonucleoprotein complex binding
	GO:0009631	0.000445880	cold acclimation
	GO:0031428	0.000447993	box C/D snoRNP complex
	GO:0080156	0.000473193	mitochondrial mRNA modification
	GO:0005635	0.000489425	nuclear envelope
	GO:0006360	0.000499518	transcription from RNA polymerase I promoter
Module 8	GO:0003964	0.000000000	RNA-directed DNA polymerase activity
	GO:0015074	0.000000000	DNA integration
	GO:0004190	0.000000000	aspartic-type endopeptidase activity
	GO:0006310	0.000000000	DNA recombination
	GO:0003887	0.000000000	DNA-directed DNA polymerase activity
	GO:0004519	0.000000000	endonuclease activity
	GO:0009507	0.000000000	chloroplast
	GO:0000943	0.000000000	retrotransposon nucleocapsid
	GO:0003676	0.000000000	nucleic acid binding
	GO:0004523	0.000000000	RNA-DNA hybrid ribonuclease activity
	GO:0032197	0.000000000	transposition, RNA-mediated
	GO:0004540	0.000000000	ribonuclease activity
	GO:0008233	0.000000000	peptidase activity
	GO:0009570	0.000000000	chloroplast stroma
	GO:0003723	0.000000000	RNA binding
	GO:0008270	0.000000000	zinc ion binding
	GO:0009941	0.000000002	chloroplast envelope
	GO:0046872	0.000001134	metal ion binding
	GO:0006313	0.000007897	transposition, DNA-mediated
	GO:0009535	0.000014685	chloroplast thylakoid membrane
	GO:0004803	0.000016409	transposase activity
Module 9	GO:0006310	0.000000003	DNA recombination
	GO:0003964	0.000000007	RNA-directed DNA polymerase activity
	GO:0004519	0.000000137	endonuclease activity
	GO:0006468	0.000002684	protein phosphorylation
	GO:0046872	0.000005693	metal ion binding
	GO:0004674	0.000021446	protein serine/threonine kinase activity
Module 10	GO:0016021	0.000000000	integral component of membrane
	GO:0006355	0.000000001	regulation of transcription, DNA-templated
	GO:0005515	0.000000002	protein binding
	GO:0003700	0.000000033	DNA binding transcription factor activity

Table 3: Gene Ontology terms enriched in each co-expression module.

Module	Category	Overrepresented p-value	Term
	GO:0006970	0.000000277	response to osmotic stress
	GO:0005794	0.000001711	Golgi apparatus
	GO:0005886	0.000002640	plasma membrane
	GO:0016020	0.000006302	membrane
	GO:0043565	0.000006311	sequence-specific DNA binding
	GO:0007275	0.000007709	multicellular organism development
	GO:0042285	0.000027768	xylosyltransferase activity
	GO:0072583	0.000031484	clathrin-dependent endocytosis
	GO:0006468	0.000040283	protein phosphorylation
	GO:0015031	0.000043071	protein transport
Module 11	GO:0043531	0.000000000	ADP binding
	GO:0009626	0.000000000	plant-type hypersensitive response
	GO:0005524	0.000000010	ATP binding
	GO:0009870	0.000000061	defense response signaling pathway, resistance gene-dependent
	GO:0006952	0.000000321	defense response
	GO:0060548	0.000003589	negative regulation of cell death
	GO:0009507	0.000005396	chloroplast
	GO:0033201	0.000012063	alpha-1,4-glucan synthase activity
	GO:0009535	0.000015676	chloroplast thylakoid membrane
	GO:0009011	0.000022960	starch synthase activity
Module 12	GO:0005886	0.000000001	plasma membrane
	GO:0006952	0.000000001	defense response
	GO:0043531	0.000000004	ADP binding
	GO:0016021	0.000000171	integral component of membrane
Module 16	GO:0009535	0.000000000	chloroplast thylakoid membrane
	GO:0015979	0.000000000	photosynthesis
	GO:0009522	0.000000000	photosystem I
	GO:0009507	0.000000000	chloroplast
	GO:0018298	0.000000000	protein-chromophore linkage
	GO:0009523	0.000000000	photosystem II
	GO:0009538	0.000000007	photosystem I reaction center
	GO:0016168	0.000000024	chlorophyll binding
	GO:0009739	0.000000078	response to gibberellin
	GO:0009789	0.000000085	positive regulation of abscisic acid-activated signaling pathway
	GO:0010598	0.000000126	NAD(P)H dehydrogenase complex (plastoquinone)
	GO:0003700	0.000000241	DNA binding transcription factor activity
	GO:0009772	0.000000342	photosynthetic electron transport in photosystem II
	GO:0070413	0.000000400	trehalose metabolism in response to stress
	GO:0009723	0.000000422	response to ethylene
	GO:0009416	0.000000441	response to light stimulus
	GO:0010319	0.000001268	stromule
	GO:0009767	0.000001277	photosynthetic electron transport chain
	GO:0010287	0.000001283	plastoglobule
	GO:0042651	0.000001324	thylakoid membrane
	GO:0007623	0.000001819	circadian rhythm
	GO:0009768	0.000002043	photosynthesis, light harvesting in photosystem I
	GO:0009773	0.000002071	photosynthetic electron transport in photosystem I

Table 3: Gene Ontology terms enriched in each co-expression module.

Module	Category	Overrepresented p-value	Term
	GO:0090229	0.000002188	negative regulation of red or far-red light signaling pathway
	GO:0009409	0.000006625	response to cold
	GO:0006355	0.000012645	regulation of transcription, DNA-templated
	GO:0005992	0.000012767	trehalose biosynthetic process
	GO:0009737	0.000019672	response to abscisic acid
	GO:0009640	0.000019796	photomorphogenesis
	GO:0046524	0.000021765	sucrose-phosphate synthase activity
	GO:0048038	0.000029880	quinone binding
	GO:0031969	0.000039092	chloroplast membrane
	GO:0009882	0.000041604	blue light photoreceptor activity
	GO:0009579	0.000043182	thylakoid
	GO:0080006	0.000129885	internode patterning
	GO:0009941	0.000136129	chloroplast envelope
	GO:0016311	0.000137838	dephosphorylation
	GO:1902448	0.000173188	positive regulation of shade avoidance
	GO:0009735	0.000176429	response to cytokinin
	GO:0019684	0.000211381	photosynthesis, light reaction

We created a Word Cloud representation using a word frequency greater than one in each enriched module to check the most common words (Figure 7).

We used the Gene Set Enrichment Analysis (GSEA) and permuted the genes of the modules 10,000 times in the ranked LFC lists of the following contrasts: i) Low against high biomass; ii) US85-1008 compared to the mean of SES205A and IN84-58; iii) White Transparent compared to the mean of RB72454 and SP80-3280. We found that module 16 was enriched with genes with high absolute LFC values in the three contrasts (Figure 9). Genes in module 16 were positivelly correlated with genes of high LFC in the biomass contrast and in the comparison of US85-1008 with two *S. spontaneum* genotypes. Ranked genes from the contrast comparing the *S. officinarum* White Transparent to the hybrids were negativelly correlated with genes within module 16 .

To visualize the expression profile of each module, we assessed the expression level of the eigengenes (Figure 10). We observed that at least five modules were marked by a expression peak or valley for a single genotype. Module 16 contains genes with higher expression in sucrose-rich genotypes, opposite to module 3. In both cases US85-1008 was in the high expression group. The profile of the eigengene of Module 16 indicates a higher expression in the low biomass group, but without a substantial variability among the samples within the group. According to the GSEA, in this module the low-biomass genotypes did not contain genes with high LFC.

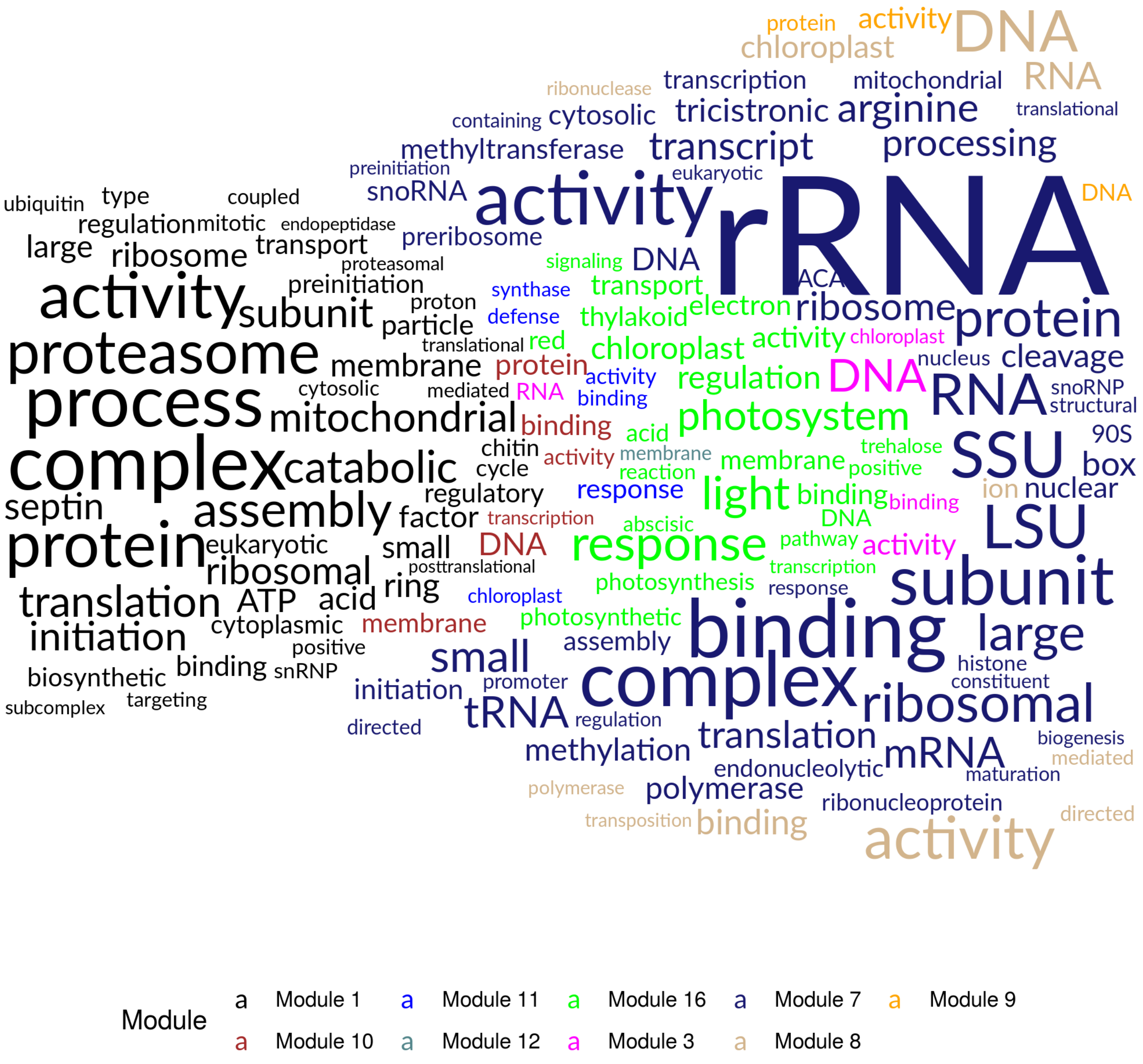

Figure 7: **Word cloud using Gene Ontology descriptions for terms enriched with genes in the co-expression modules.** This image is restricted to present only nine modules, those that presented enriched GO terms.

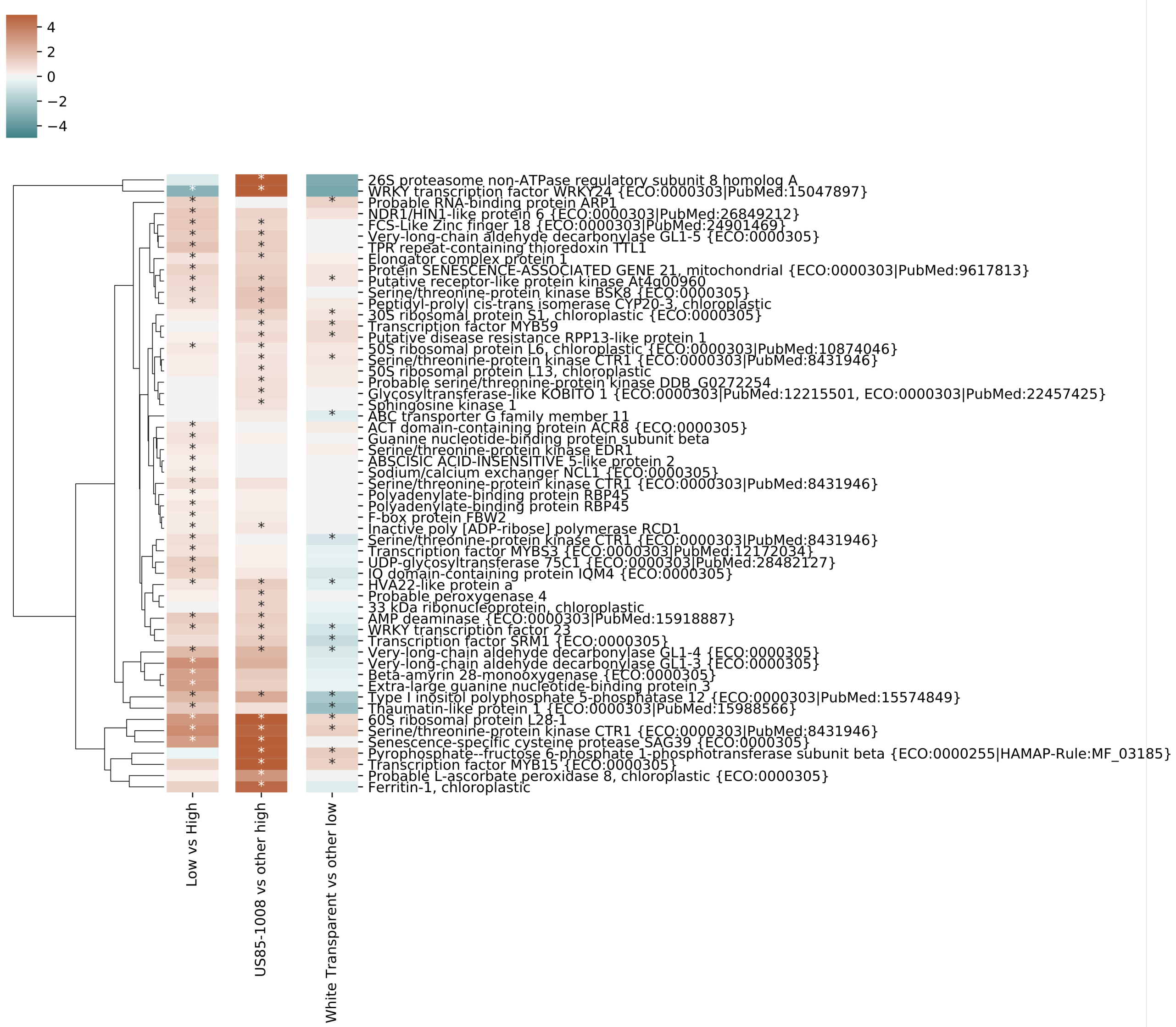

Figure 8: **Expression of hormone response DEGs present in the co-expression module 16** These genes were functionally annotated to the biological processes of responses to abscisic acid, cytokinin, ethylene or gibberellin. Each column in the heatmap represent the genes fold changes according to a contrast: (i) *Low vs High* is the comparison between the low biomass to the high biomass group; (ii) *US85-1008 vs other high* represents the comparison of US85-1008 to wild high-fiber canes; (iii) *White Transparent vs other low* is the contrast of White Transparent against the low-fiber hybrids. Significantly differentially expressed genes are indicated by asterisks.

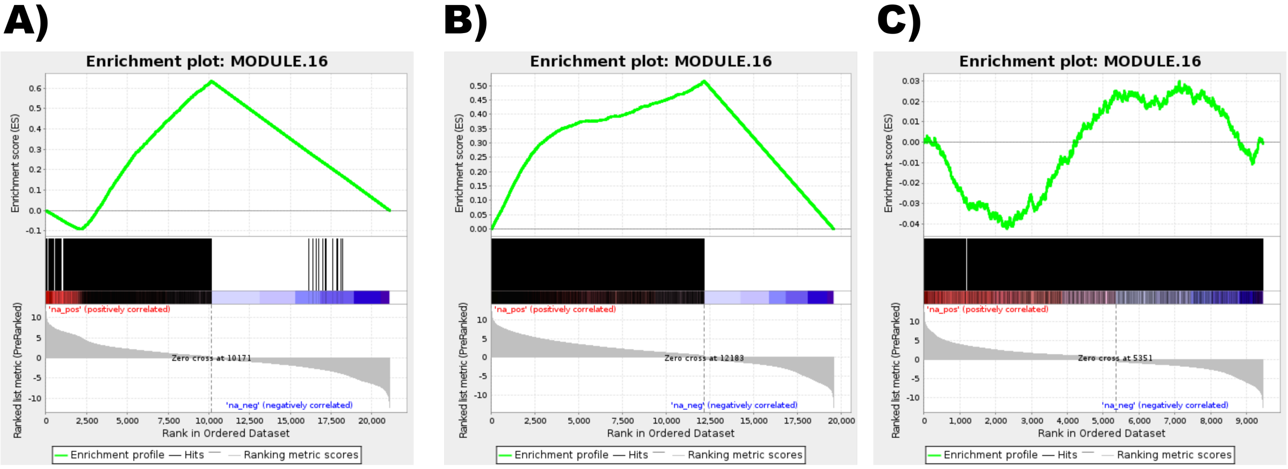

Figure 9: **Gene Set Enrichment of the Module 16 co-expressed genes** Gene set enrichment using genes ranked based on absolute LFC. (A) Low-fiber genotypes contrasted to the high-fiber group. (B) US85-1008 contrasted to the mean of SES205A and IN84-58. (C) White Transparent compared to the mean of the hybrids RB72454 and SP80-3280

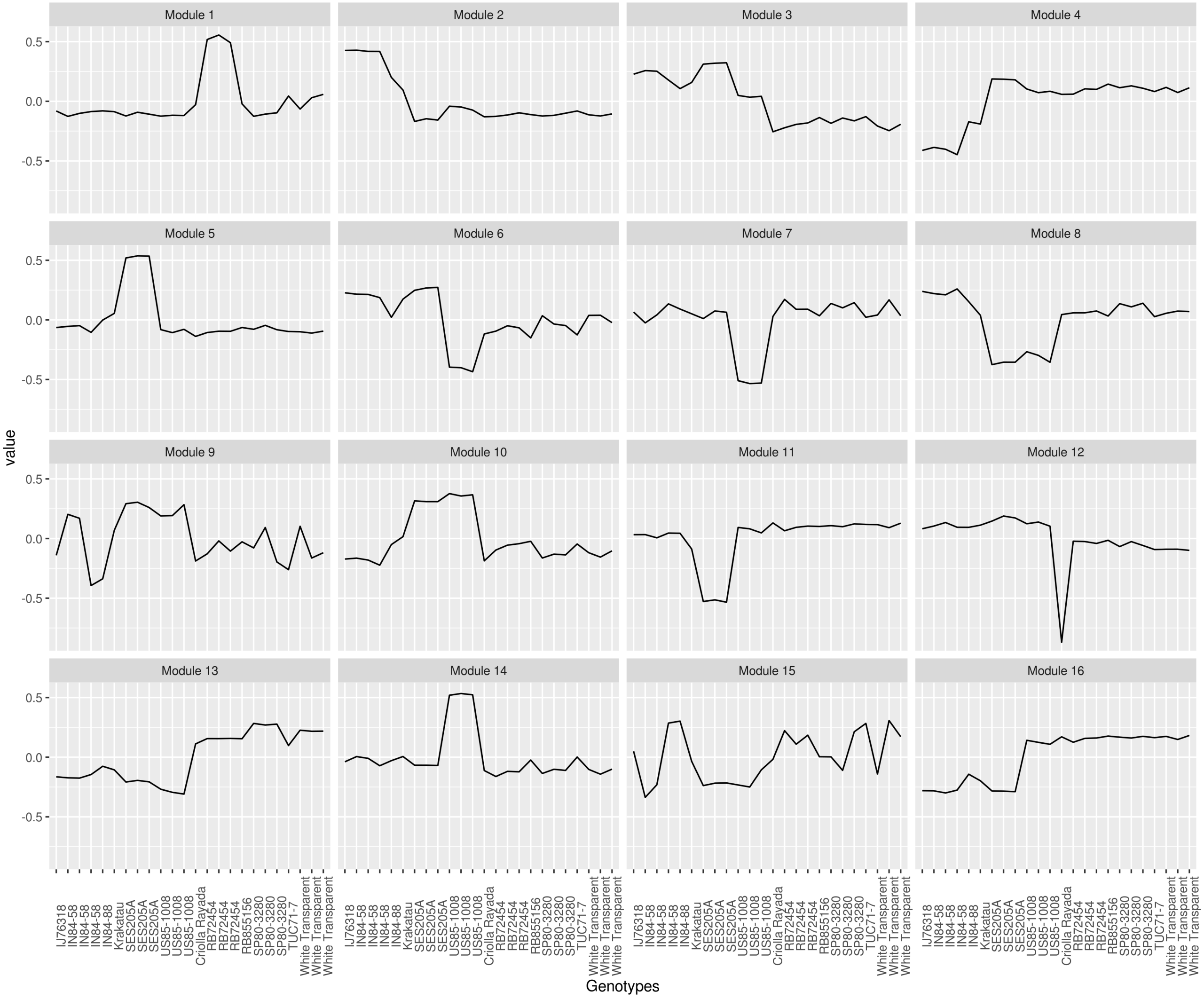

Figure 10: **Expression profile of the eigengenes from each module.**

4 Pathway analysis with Gene Ontology terms and MapMan4

We explored the pathways provided by MapMan4 to associate up and downregulated DEGs with metabolic processes. We first used all the isoforms of a gene to map to the functional annotation BINs in Mercator4. Next, in MapMan we used the log fold change of the DEGs identified in each contrast evaluated. Here we present the results of the *metabolism overview* and *lignin* pathways.

4.1 Metabolism overview

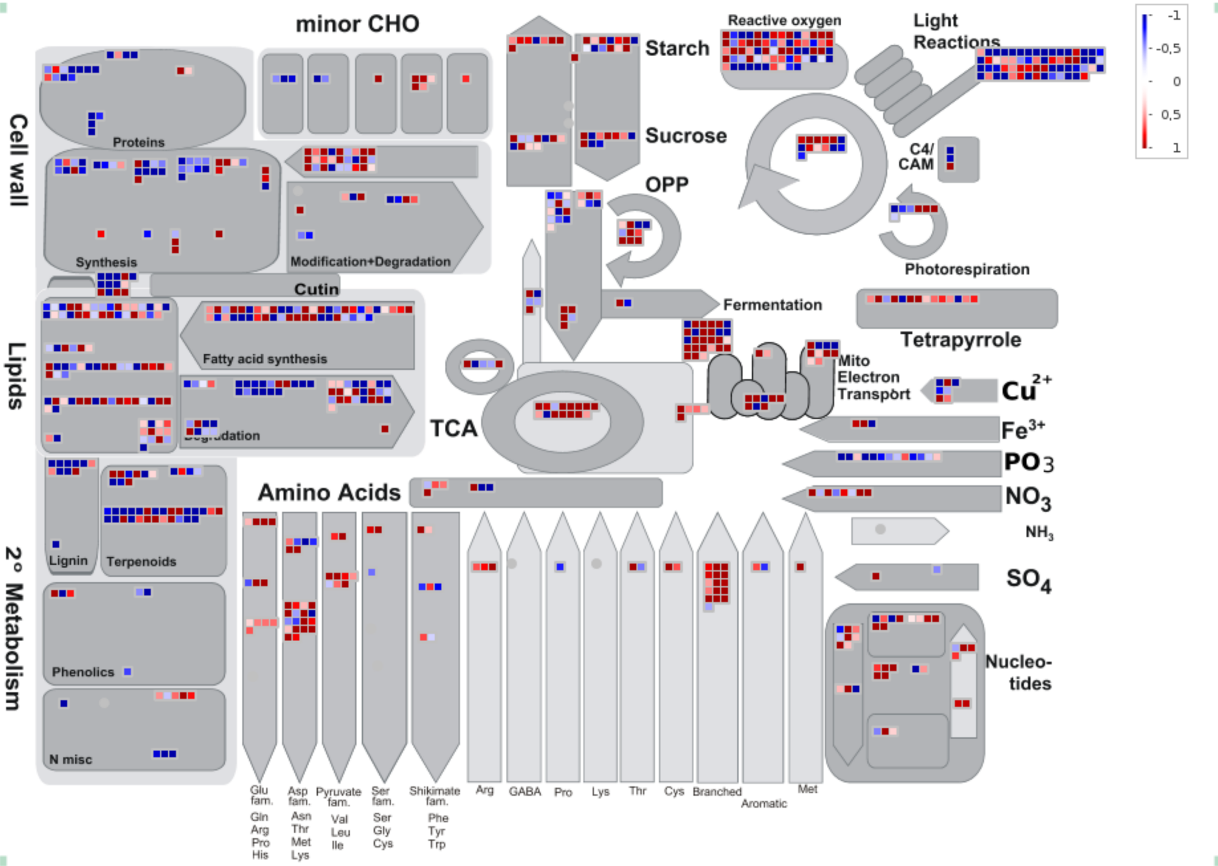

Figure 11: Metabolism overview mapping using the log of fold change of the DEGs from the low biomass genotypes compared to the high biomass group. Genes significantly upregulated were colored in red, while those downregulated were colored in blue.

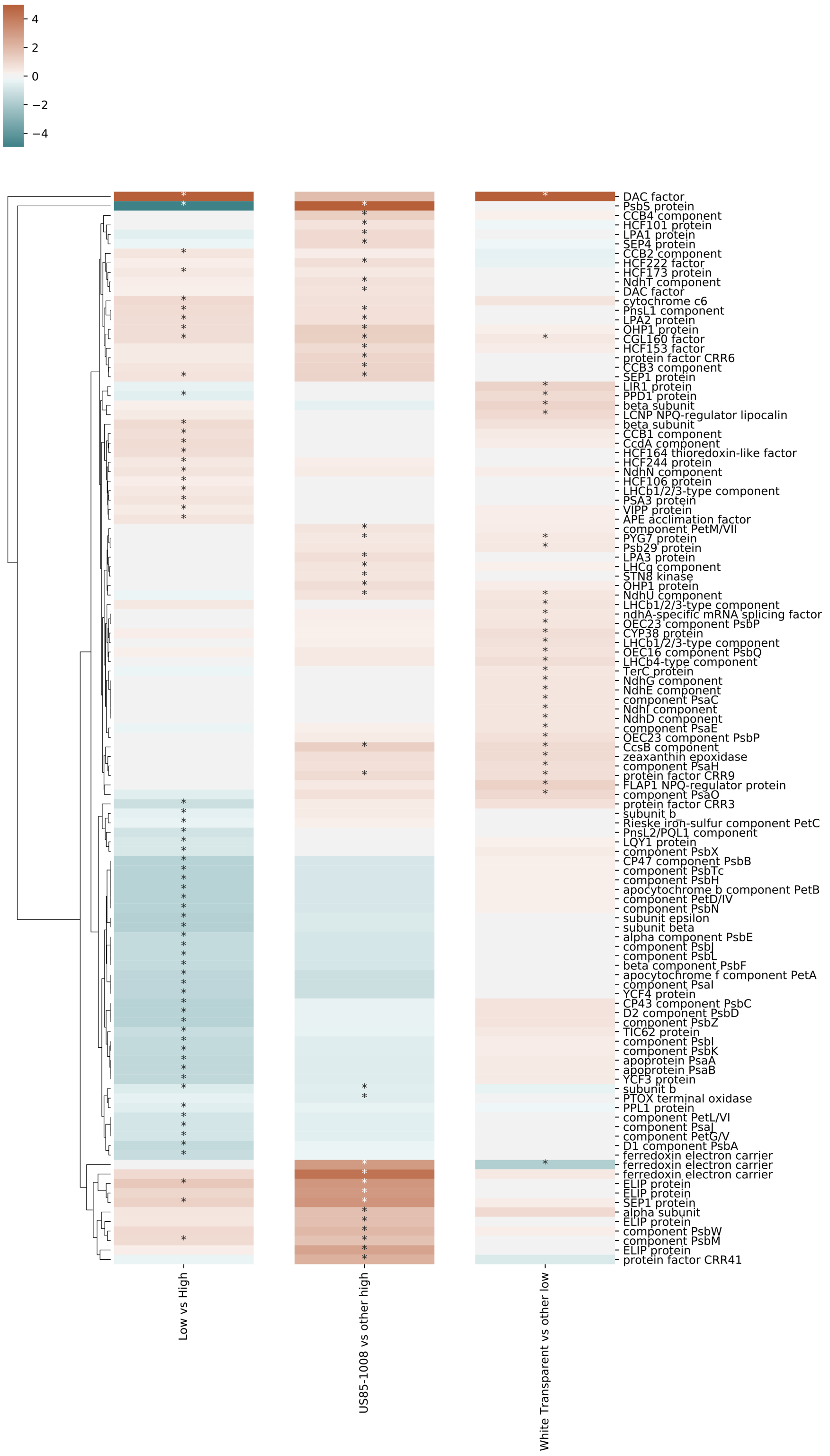

Figure 12: **Heatmap for MapMan Photophosphorylatyon annotation.** Each column in the heatmap represent the genes fold changes according to a contrast: (i) *Low vs High* is the comparison between the low biomass to the high biomass group; (ii) *US85-1008 vs other high* represents the comparison of US85-1008 to wild high-fiber canes; (iii) *White Transparent vs other low* is the contrast of White Transparent against the low-fiber hybrids. Differentially expressed genes of the contrast are indicated by asterisks.

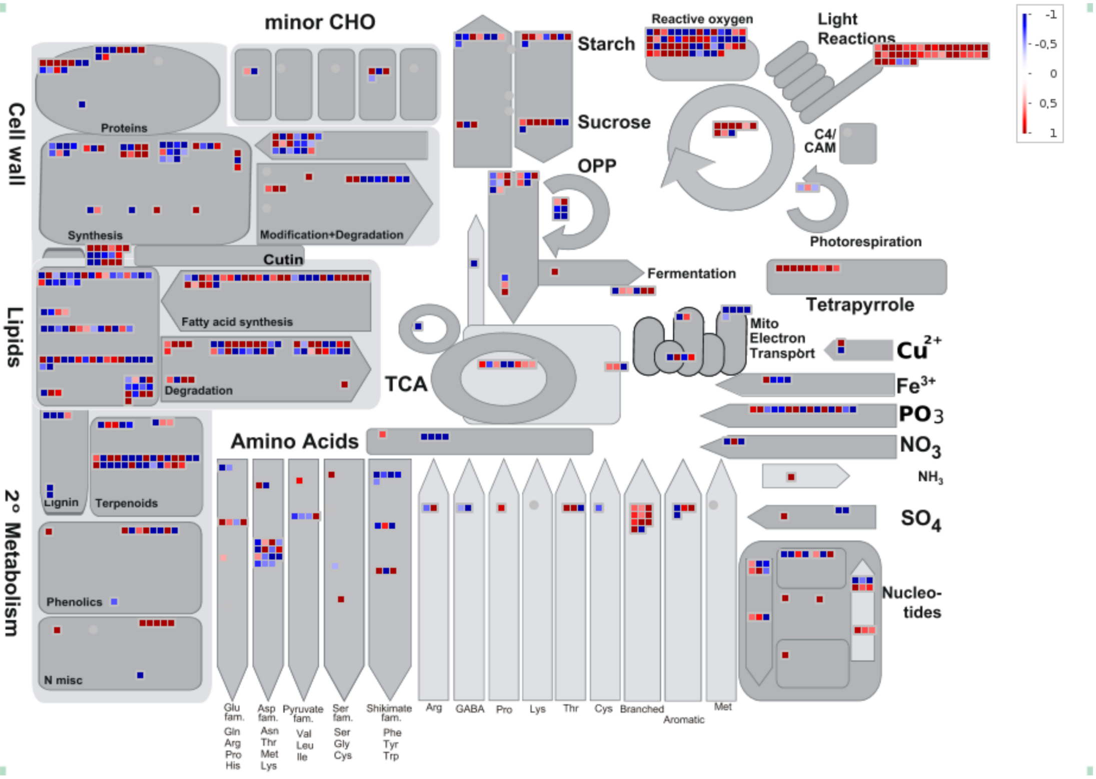

Figure 13: Metabolism overview mapping using the log of fold change of the DEGs from the comparison between US85-1008 and the mean of SES205A and IN84-58. Genes upregulated in US85-1008 were colored in red and those downregulated were colored in blue.

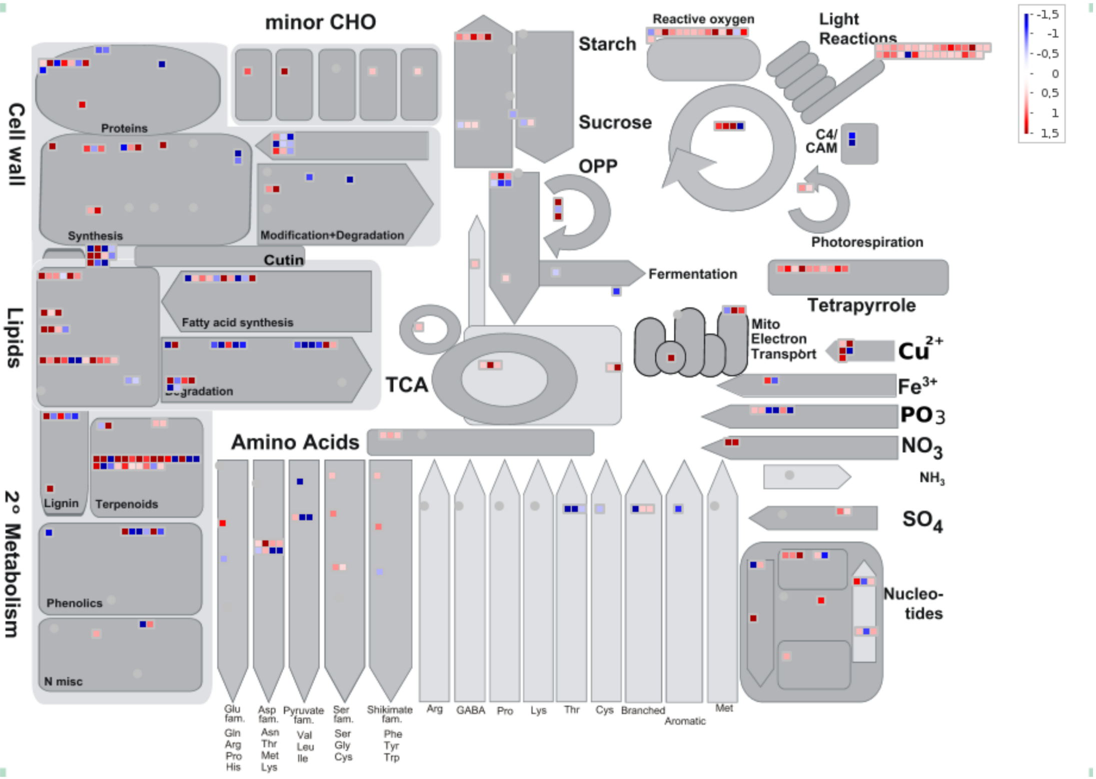

Figure 14: Metabolism overview mapping using the log of fold change of the DEGs from the comparison between White Transparent and the mean of RB72454 and SP80-3280. Genes upregulated in White Transparent were colored in red and those downregulated were colored in blue.

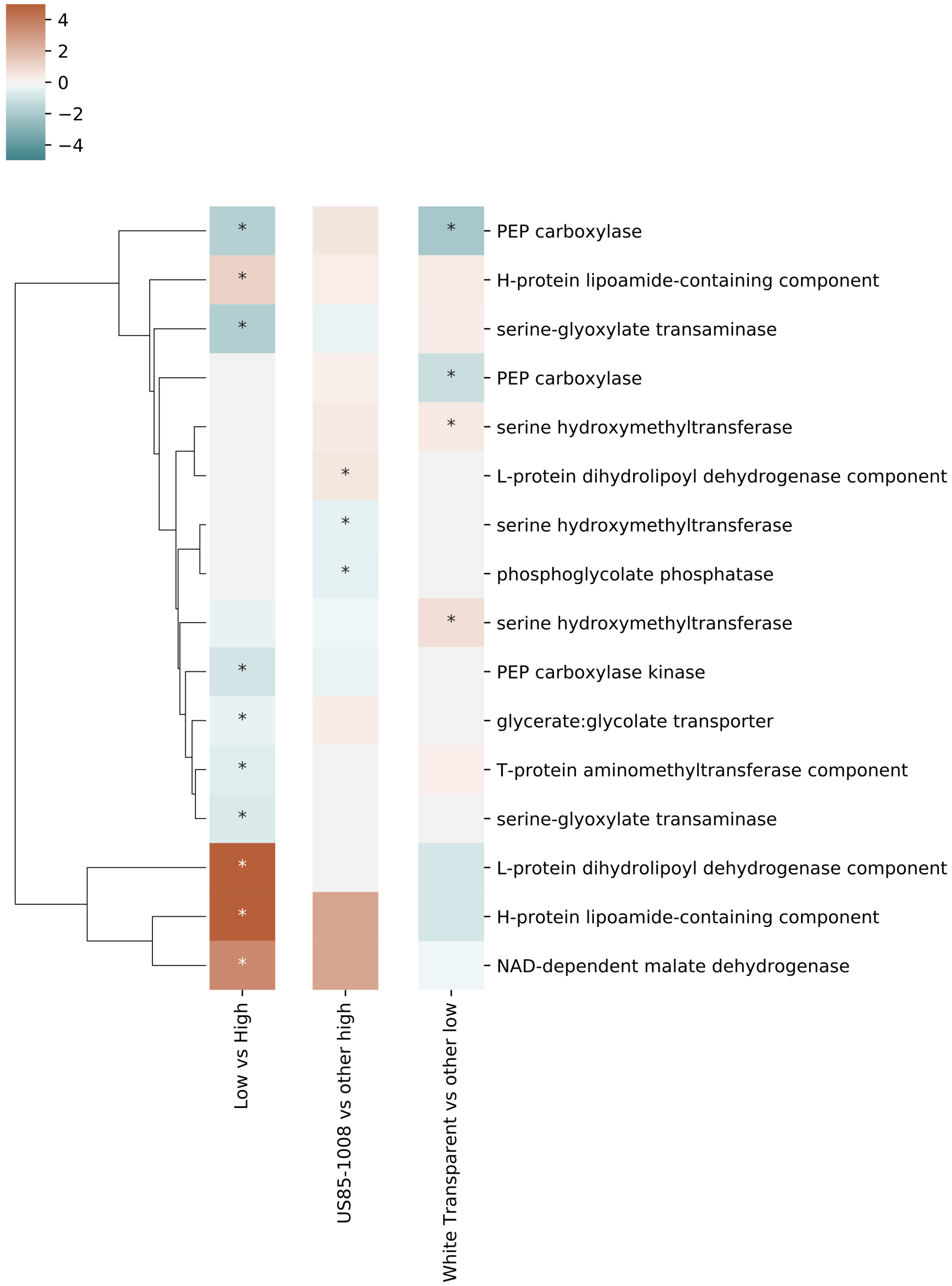

Figure 15: **Heatmap for MapMan C4/CAM photosynthesis and photorespiration annotations.** Each column in the heatmap represent the genes fold changes according to a contrast: (i) *Low vs High* is the comparison between the low biomass to the high biomass group; (ii) *US85-1008 vs other high* represents the comparison of US85-1008 to wild high-fiber canes; (iii) *White Transparent vs other low* is the contrast of White Transparent against the low-fiber hybrids. Differentially expressed genes of the contrast are indicated by asterisks.

4.2 Lignin

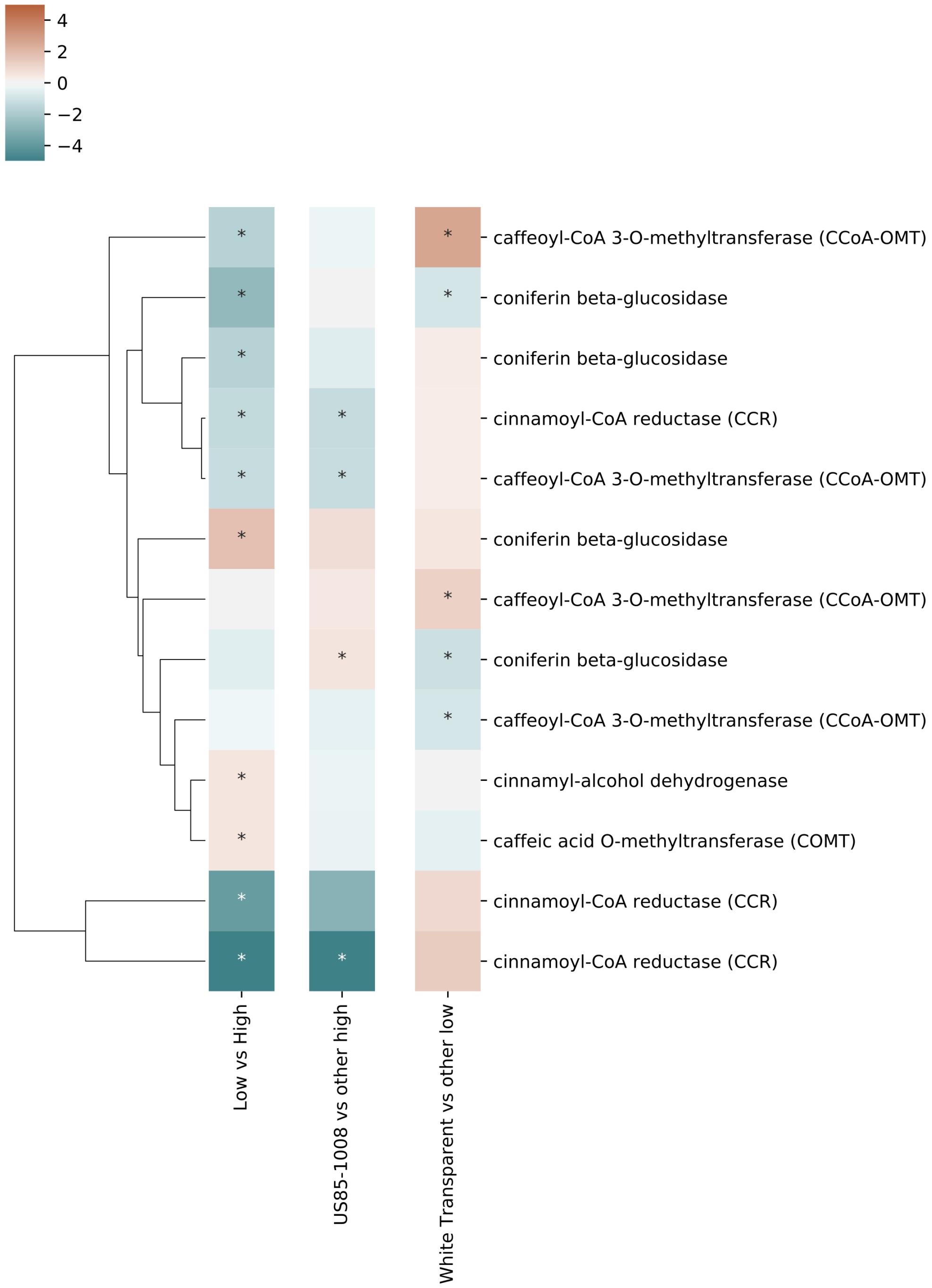

Figure 16: **Heatmap for MapMan monolignol synthesis and monolignol glycosylation and deglycosylation.** Each column in the heatmap represent the genes fold changes according to a contrast: (i) *Low vs High* is the comparison between the low biomass to the high biomass group; (ii) *US85-1008 vs other high* represents the comparison of US85-1008 to wild high-fiber canes; (iii) *White Transparent vs other low* is the contrast of White Transparent against the low-fiber hybrids. Differentially expressed genes of the contrast are indicated by asterisks.

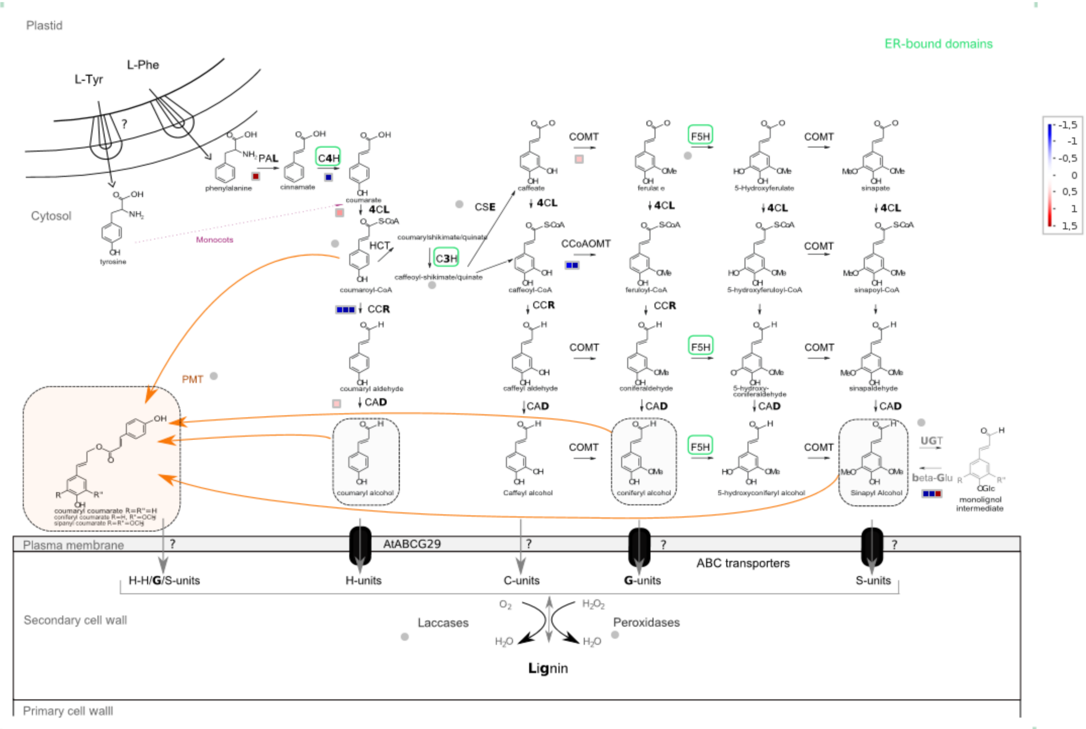

Figure 17: Lignin pathway mapping using the log of fold change of the DEGs from the low biomass genotypes compared to the high biomass group. Genes significantly upregulated were colored in red, while those downregulated were colored in blue.

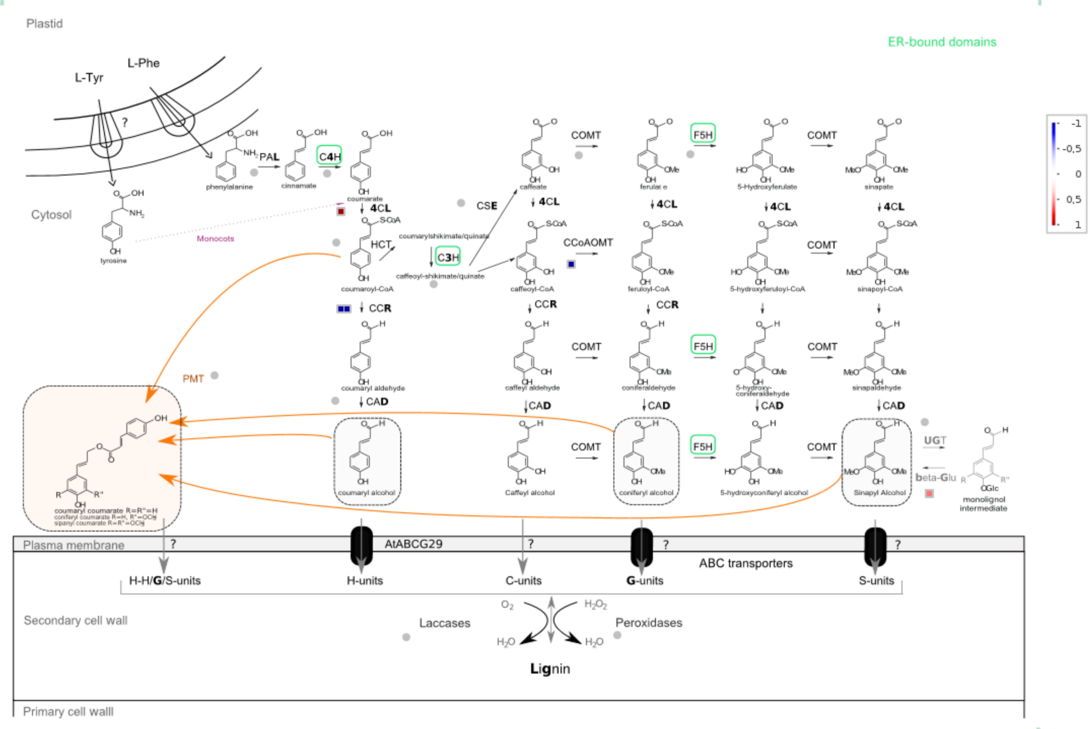

Figure 18: Lignin pathway mapping using the log of fold change of the DEGs from the comparison between US85-1008 and the mean of SES205A and IN84-58. Genes upregulated in US85-1008 were colored in red and those downregulated were colored in blue.

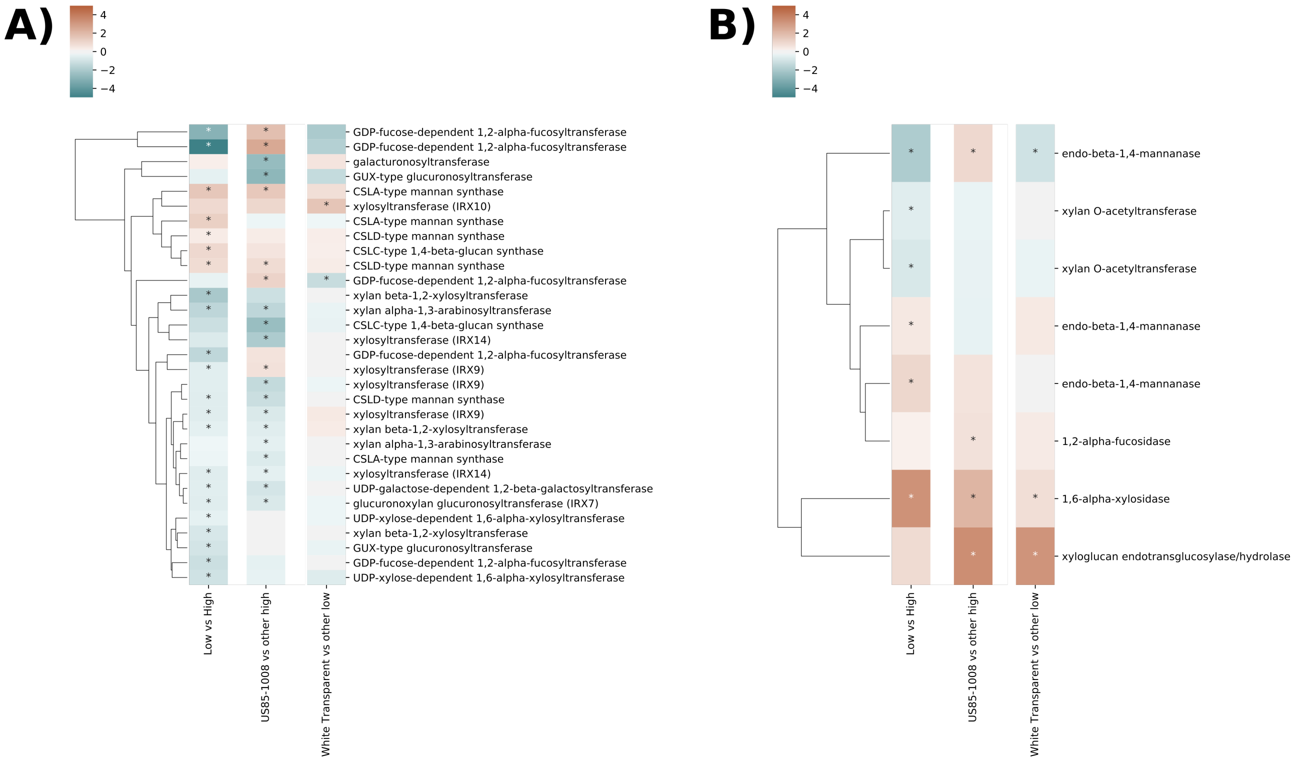

Figure 19: **Heatmap for MapMan synthesis (A) and modification and degradation (B) of the cell wall compounds.** Each column in the heatmap represent the genes fold changes according to a contrast: (i) *Low vs High* is the comparison between the low biomass to the high biomass group; (ii) *US85-1008 vs other high* represents the comparison of US85-1008 to wild high-fiber canes; (iii) *White Transparent vs other low* is the contrast of White Transparent against the low-fiber hybrids. Differentially expressed genes of the contrast are indicated by asterisks.

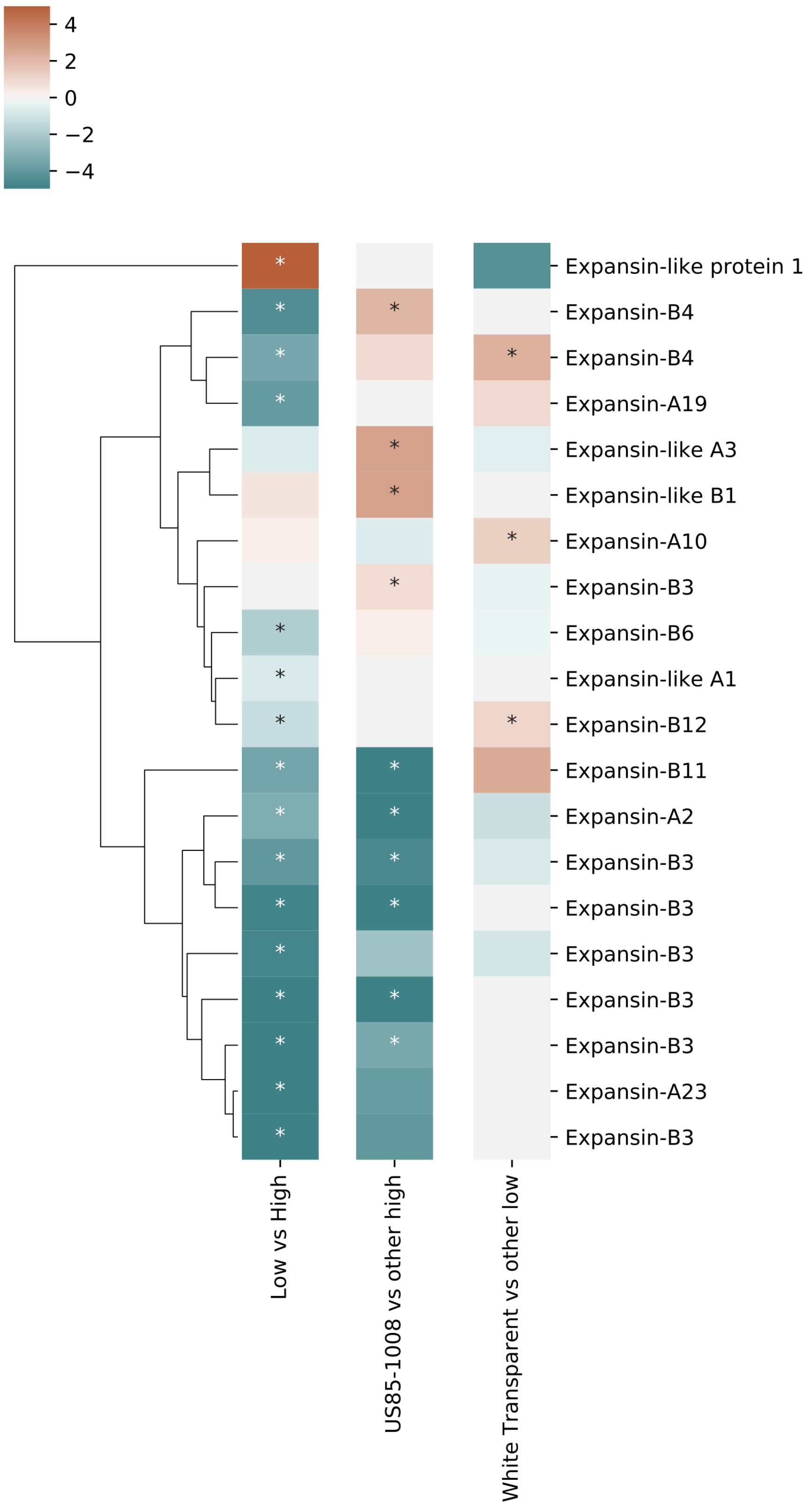

Figure 20: **Expression of DEGs coding for expansins.** Each column in the heatmap represent the genes fold changes according to a contrast: (i) *Low vs High* is the comparison between the low biomass to the high biomass group; (ii) *US85-1008 vs other high* represents the comparison of US85-1008 to wild high-fiber canes; (iii) *White Transparent vs other low* is the contrast of White Transparent against the low-fiber hybrids. Differentially expressed genes of the contrast are indicated by asterisks.

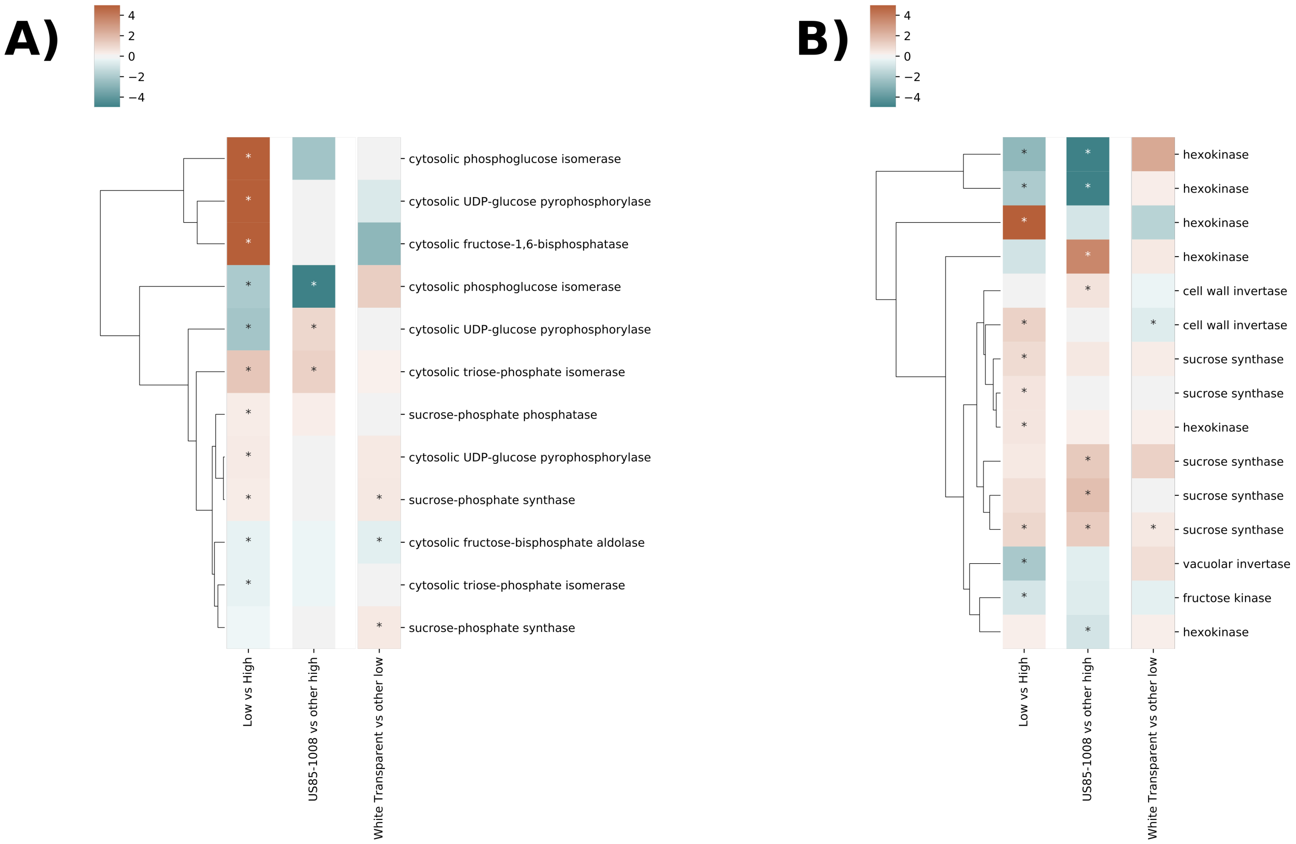

Figure 21: **Heatmap for MapMan sucrose metabolism of synthesis (A) and degradation (B).** Each column in the heatmap represent the genes fold changes according to a contrast: (i) *Low vs High* is the comparison between the low biomass to the high biomass group; (ii) *US85-1008 vs other high* represents the comparison of US85-1008 to wild high-fiber canes; (iii) *White Transparent vs other low* is the contrast of White Transparent against the low-fiber hybrids. Differentially expressed genes of the contrast are indicated by asterisks.

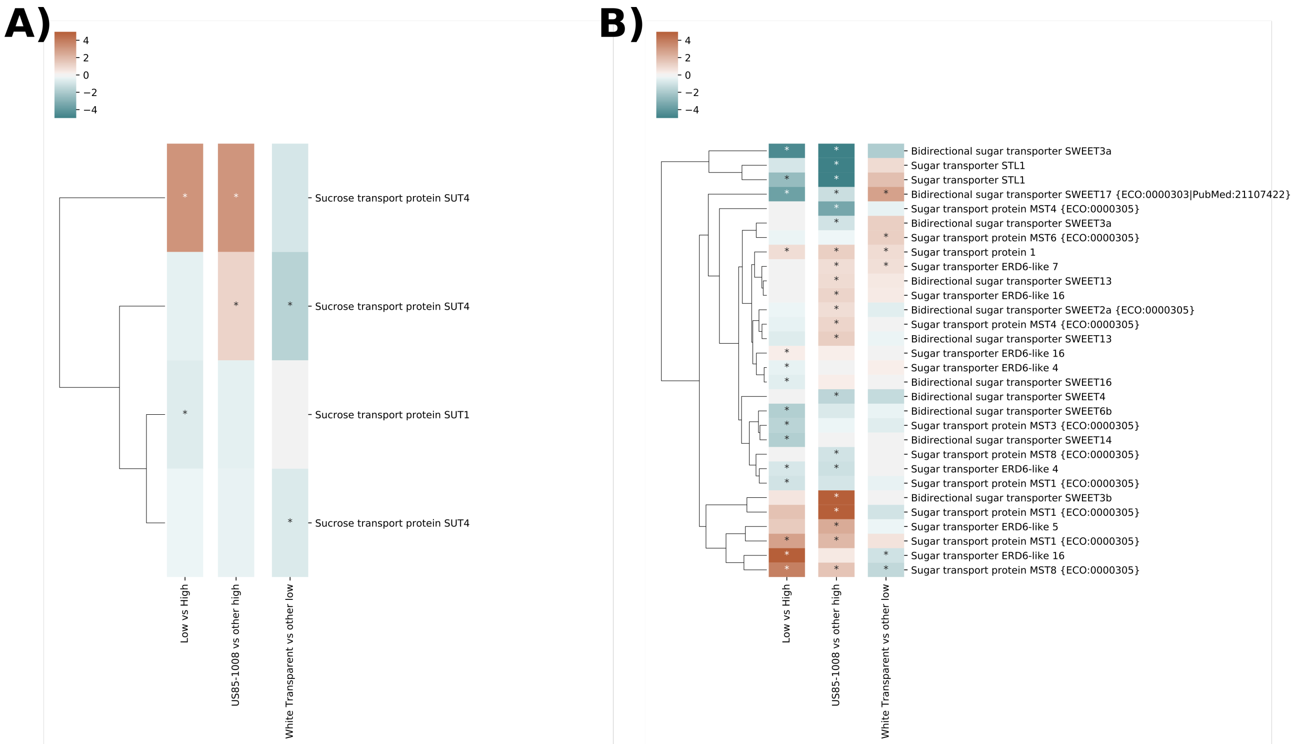

Figure 22: **Expression of DEGs coding for sucrose transport proteins (A) and sugar transporters (B).** Each column in the heatmap represent the genes fold changes according to a contrast: (i) *Low vs High* is the comparison between the low biomass to the high biomass group; (ii) *US85-1008 vs other high* represents the comparison of US85-1008 to wild high-fiber canes; (iii) *White Transparent vs other low* is the contrast of White Transparent against the low-fiber hybrids. Differentially expressed genes of the contrast are indicated by asterisks.

4.3 Expression of investigated genes

In this subsection we present the log of counts per million (logCPM) in the three contrasts for genes investigated by their biological relevance. Expression of four genes encoding sucrose synthase (SuSy) are in Table 4.

Table 4: Expression of sucrose synthase genes. The expression levels, in logCPM, were obtained from the contrast comparing the two phenotypically distinct groups and from the contrast within each group. Asterisk indicates if the gene is differentially expressed in the contrast.

	**Low fiber vs High fiber**	**US85-1008 vs other high fiber**	***S. officinarum* vs other low fiber**
trinity_dn11006_c0_g1	3.659	3.047*	3.829
trinity_dn11963_c0_g1	6.04*	5.692	6.317
trinity_dn141746_c0_g1	-0.338	-0.355*	-0.473
trinity_dn14183_c0_g1	1.672*	1.262	2.048
trinity_dn931_c0_g1	6.833*	6.412*	7.186*

References

[1] Rob Patro, Geet Duggal, Michael I Love, Rafael A Irizarry, and Carl Kingsford. Salmon: fast and bias-aware quantification of transcript expression using dual-phase inference. *Nature Methods*, 14(4):417–419, 2017.
