# Supplementary material for: Differential expression in leaves of Saccharum genotypes contrasting in biomass production provides evidence of genes involved in carbon partitioning
Source: BMC Genomics. 2020 Sep 29;21:673. doi: 10.1186/s12864-020-07091-y (PMC7526157; doi:10.1186/s12864-020-07091-y)
Supplement: Supplementary file 4 — Additional file 4. Supporting information for differentially expressed transcripts. Additional tables and figures about the analyses comparing the expression of genes and transcripts. [file 12864_2020_7091_MOESM4_ESM.pdf]

S4 - Supporting information for differentially expressed transcripts

1 Comparing the expression of genes and transcripts

We checked the similarity of results when the quantification was done for the whole transcriptome, considering each individual isoform, or when we grouped expression levels at gene-level. The number of expressed genes and transcripts can be found on Table 1. In both cases, the number represents roughly 30% of the complete reference used.

	Number passing the expression filter	Percentage of total reference
Genes	47676	0.27
Transcripts	133232	0.30

Table 1: Number of genes and transcripts kept after minimum expression filter for each of the quantification methods.

We also compared if the differentially expressed transcripts (DETs) corresponded to the differentially expressed genes (DEGs) when the analysis was performed grouping counts at the gene level. First, we used the contrast comparing biomass groups. The number of DEGs and DETs can be seen on Table 2. For 15,188 DEGs, at least one of its transcripts was differentially expressed (Figure 1).

	Genes	Transcripts
Down	10903	21996
Not DE	26602	90850
Up	10171	20386

Table 2: Number of downregulated (Down), not differentially expressed (Not DE) and upregulated (Up) genes or transcripts .

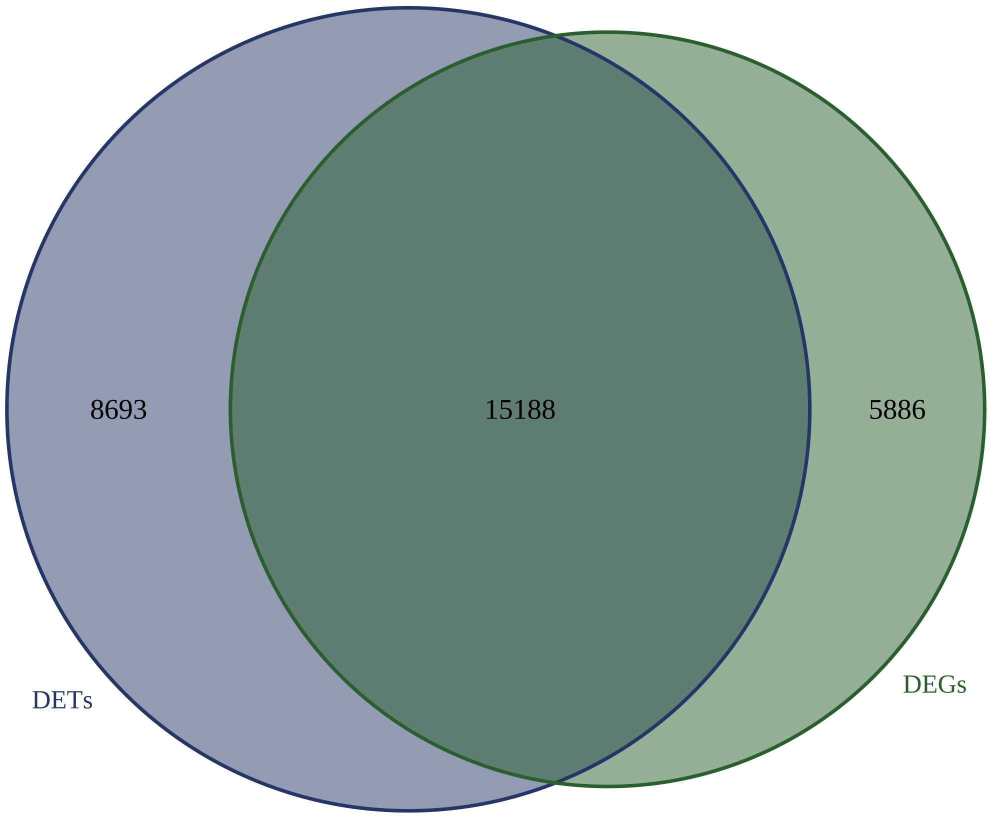

Figure 1: Venn Diagram showing the overlap between the list of differentially expressed genes and differentially expressed transcripts.

We observed that the same six processes enriched among DEGs were also enriched in the DETs (Table 3). Analysis of DETs showed enrichment of terms related to transposition, defense response and enzymatic activities. Enriched terms related to *photosynthesis* (*glyceraldehyde-3-phosphate dehydrogenase (NAD+) (phosphorylating) activity* and *geranylgeranyl-diphosphate geranylgeranyltransferase activity*) that carrying out differential expression analysis of isoforms revealed more specific processes. In the case of *glyceraldehyde-3-phosphate dehydrogenase* (GADPH), we noted that only two non-DEGs presented DETs (Figure 4).

We focused the search of DEGs and DETs to a group of annotated genes with a common gene ontology term. We chose the *photosyntesis* biological process as an example. Only 18 DEGs had at least one isoform as differentially expressed, 12 genes found as differentially expressed did not have DETs and 47 genes had differentially expressed isoforms but were not differentially expressed when counts were grouped into the gene level (Figure 2). Five photosynthesis DEGs did not have at least one DET (Figure 3).

Category	Description	Genes	DEGs	Transcripts	DETs	Enriched DEGs	Enriched DETs
GO:0002181	cytoplasmic translation	81	58	129	59	Yes	Yes
GO:0003964	RNA-directed DNA polymerase activity	1212	592	1841	712	Yes	Yes
GO:0004190	aspartic-type endopeptidase activity	1045	500	1743	649	Yes	Yes
GO:0004519	endonuclease activity	945	457	1526	557	Yes	Yes
GO:0004523	RNA-DNA hybrid ribonuclease activity	653	326	878	357	Yes	Yes
GO:0015074	DNA integration	986	474	1562	592	Yes	Yes
GO:0000943	retrotransposon nucleocapsid	768	344	1217	423	No	Yes
GO:0003676	nucleic acid binding	1857	834	4312	1334	No	Yes
GO:0003887	DNA-directed DNA polymerase activity	384	179	626	233	No	Yes
GO:0004310	farnesyl-diphosphate farnesyltransferase activity	7	3	48	25	No	Yes
GO:0004365	glyceraldehyde-3-phosphate dehydrogenase (NAD+) (phosphorylating) activity	16	11	55	30	No	Yes
GO:0004514	nicotinate-nucleotide diphosphorylase (carboxylating) activity	4	1	13	10	No	Yes
GO:0004540	ribonuclease activity	227	101	311	120	No	Yes
GO:0004674	protein serine/threonine kinase activity	1778	753	5026	1535	No	Yes
GO:0004803	transposase activity	108	50	244	110	No	Yes
GO:0005524	ATP binding	5497	2298	16431	4844	No	Yes
GO:0006310	DNA recombination	1090	504	1890	663	No	Yes
GO:0006313	transposition, DNA-mediated	139	73	283	125	No	Yes
GO:0006696	ergosterol biosynthetic process	19	12	67	36	No	Yes
GO:0006952	defense response	1840	765	4157	1352	No	Yes
GO:0008171	O-methyltransferase activity	57	29	164	77	No	Yes
GO:0008270	zinc ion binding	1686	699	4323	1308	No	Yes
GO:0008615	pyridoxine biosynthetic process	7	4	19	14	No	Yes
GO:0008825	cyclopropane-fatty-acyl-phospholipid synthase activity	2	1	10	9	No	Yes

Category	Description	Genes	DEGs	Transcripts	DETs	Enriched DEGs	Enriched DETs
GO:0009443	pyridoxal 5'-phosphate salvage	4	2	20	14	No	Yes
GO:0009870	defense response signaling pathway, resistance gene-dependent	225	94	452	161	No	Yes
GO:0010942	positive regulation of cell death	75	34	152	65	No	Yes
GO:0016767	geranylgeranyl-diphosphate geranylgeranyltransferase activity	6	3	37	22	No	Yes
GO:0016866	intramolecular transferase activity	33	18	88	44	No	Yes
GO:0017148	negative regulation of translation	74	39	200	81	No	Yes
GO:0019438	aromatic compound biosynthetic process	28	14	73	36	No	Yes
GO:0030598	rRNA N-glycosylase activity	41	22	83	52	No	Yes
GO:0032197	transposition, RNA-mediated	342	155	465	185	No	Yes
GO:0032199	reverse transcription involved in RNA-mediated transposition	132	62	197	78	No	Yes
GO:0032201	telomere maintenance via semi-conservative replication	3	2	7	7	No	Yes
GO:0042301	phosphate ion binding	10	8	24	17	No	Yes
GO:0043531	ADP binding	1199	517	2620	925	No	Yes
GO:0043657	host cell	9	6	18	14	No	Yes
GO:0046718	viral entry into host cell	29	17	42	26	No	Yes
GO:0046905	phytoene synthase activity	4	1	35	20	No	Yes
GO:0046983	protein dimerization activity	438	199	1245	444	No	Yes
GO:0051286	cell tip	10	9	12	10	No	Yes
GO:0051996	squalene synthase activity	7	3	48	25	No	Yes
GO:0060548	negative regulation of cell death	179	73	355	130	No	Yes
GO:0070987	error-free translesion synthesis	2	2	7	7	No	Yes
GO:0071768	mycolic acid biosynthetic process	1	0	10	9	No	Yes
GO:0075732	viral penetration into host nucleus	19	12	23	17	No	Yes
GO:0090305	nucleic acid phosphodiester bond hydrolysis	136	64	214	83	No	Yes
GO:0090729	toxin activity	30	13	65	35	No	Yes

Table 3: Gene Ontology terms enriched among DEGs or DETs using the contrast between biomass groups. The number of genes, differentially expressed genes, transcripts and differentially expressed transcripts for each GO term are shown. The last two columns indicate if the term was enriched among DEGs and DETs, respectively.

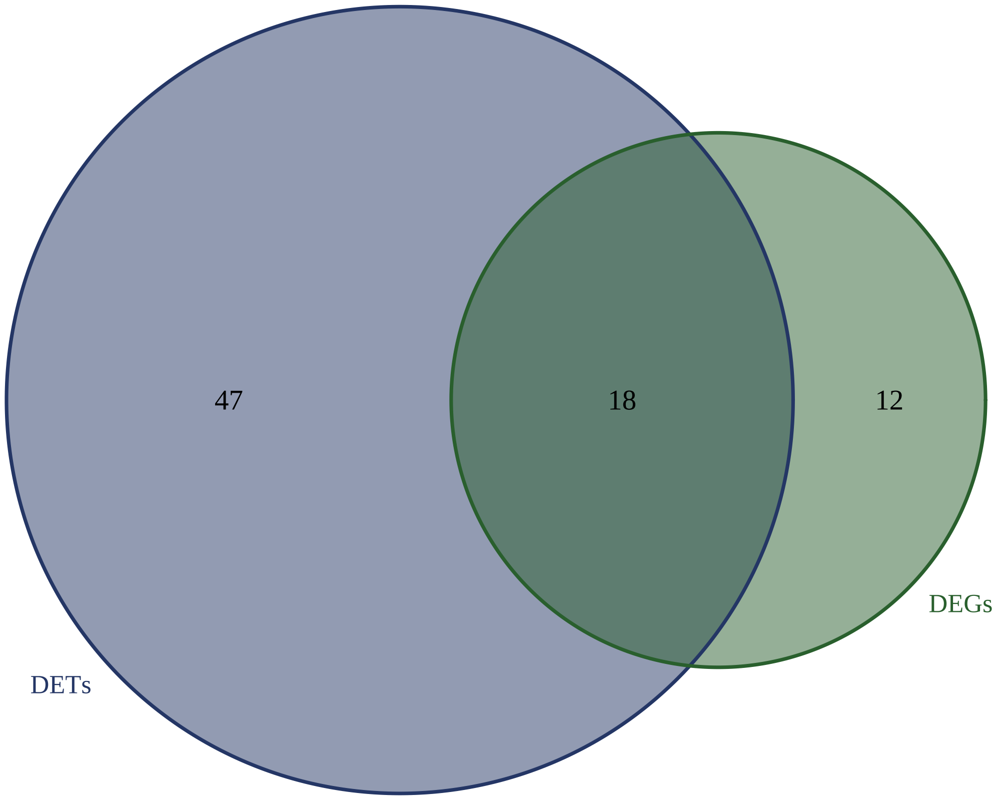

Figure 2: Venn Diagram showing overlap between the DEGs and DETs of the *photosynthesis* biological process.

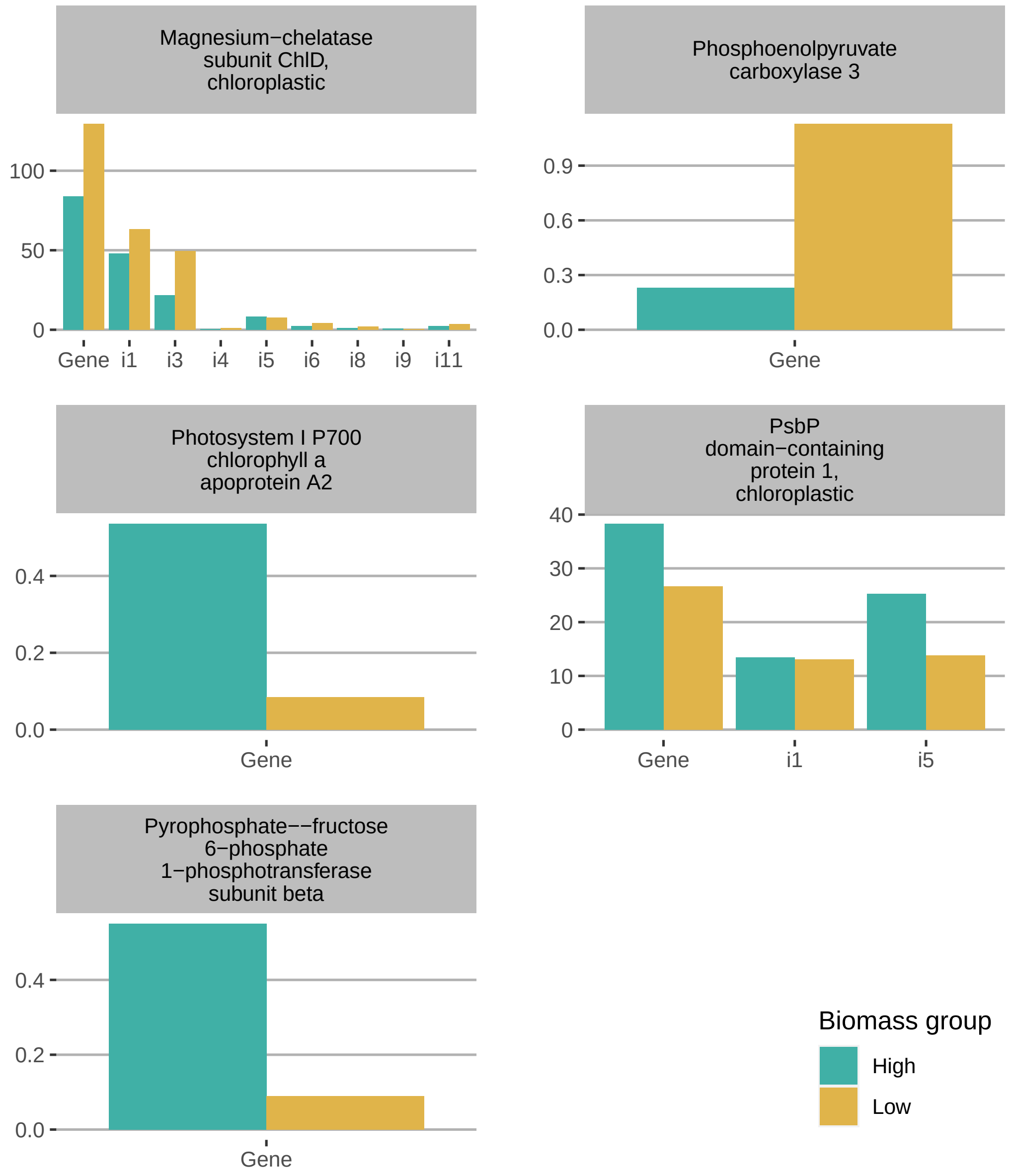

Figure 3: Genes associated to photosynthesis without differentially expressed transcripts. Expression, in counts per million, was measured for each biomass group. In the $x - axis$, transcripts passing the expression filter are shown beside the corresponding gene, indicated with the prefix **i**.

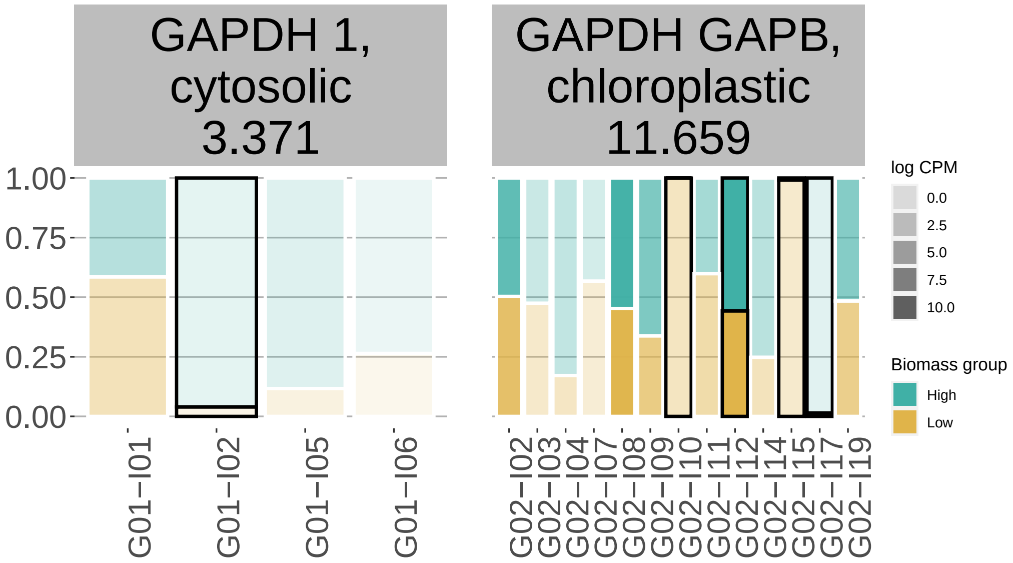

Figure 4: **Expression differentially expressed transcripts corresponding to non-differentially expressed *glyceraldehyde-3-phosphate dehydrogenase (NAD+) (phosphorylating) activity* related genes.** The identificator of each gene provides the log of the counts per million of it. For the isoforms, the measure of expression is in counts per million shown as a proportion between the biomass groups, in different colors. The intensity of the color represents the logarithm of the counts per million (log CPM) of the transcript. Differentially expressed transcripts have their edges in black.
